# Discoveries in Pancreatic Physiology and Disease Biology Using Single-Cell RNA Sequencing

**DOI:** 10.3389/fcell.2021.732776

**Published:** 2022-01-24

**Authors:** Haotian Fu, Hongwei Sun, Hongru Kong, Bin Lou, Hao Chen, Yilin Zhou, Chaohao Huang, Lei Qin, Yunfeng Shan, Shengjie Dai

**Affiliations:** ^1^ Department of Hepatobiliary and Pancreatic Surgery, The First Affiliated Hospital of Wenzhou Medical University, Wenzhou, China; ^2^ Key Laboratory of Diagnosis and Treatment of Severe Hepato-Pancreatic Diseases of Zhejiang Province, Wenzhou, China; ^3^ Department of Surgery, The Third People’s Hospital of Yuhang District, Hangzhou, China; ^4^ Department of Thyroid Surgery, The First Affiliated Hospital of Wenzhou Medical University, Wenzhou, China; ^5^ Department of Biology, Boston University, Boston, MA, United States; ^6^ Department of General Surgery, The First Affiliated Hospital of Soochow University, Suzhou, China

**Keywords:** single-cell, single-cell RNA sequencing, pancreas, diabetes, pancreatic cancer

## Abstract

Transcriptome analysis is used to study gene expression in human tissues. It can promote the discovery of new therapeutic targets for related diseases by characterizing the endocrine function of pancreatic physiology and pathology, as well as the gene expression of pancreatic tumors. Compared to whole-tissue RNA sequencing, single-cell RNA sequencing (scRNA-seq) can detect transcriptional activity within a single cell. The scRNA-seq had an invaluable contribution to discovering previously unknown cell subtypes in normal and diseased pancreases, studying the functional role of rare islet cells, and studying various types of cells in diabetes as well as cancer. Here, we review the recent *in vitro* and *in vivo* advances in understanding the pancreatic physiology and pathology associated with single-cell sequencing technology, which may provide new insights into treatment strategy optimization for diabetes and pancreatic cancer.

## Introduction

Sequencing technology is increasingly used to study the endocrine function of the pancreas under physiological and pathological conditions, as well as the driving factors of pancreatic tumor development. Whole-tissue RNA sequencing is mainly utilized to determine the main differences in gene expression under disease and normal conditions. However, it provides the average mRNA expression patterns associated with the mixed RNA signal from diverse cells within the same tissue. Hence, it is greatly affected by cell number and type, and it is difficult to study cell heterogeneity, rare cell populations, and cannot be used to analyze the associated tumor microenvironment and underlying factors of cancer development. In the precision medicine era, the characterization of complex diseases and heterogeneous tissue such as diabetes and pancreatic tumors require advanced sequencing methods.

Recent technology has made it possible to map the RNA expression of a single cell. This technology is called scRNA-seq. Since Tang et al. (2009) first measured the transcriptome at cellular level, scRNA-seq witnessed an explosive development (Tang et al., 2009; Shapiro et al., 2013; Stegle et al., 2015; Svensson et al., 2017). Compared to whole-tissue RNA-seq methods, scRNA-seq can provide considerable insights into cell heterogeneity which might carry profound implications for biology (Tang et al., 2011; Janiszewska et al., 2015; Saadatpour et al., 2015; Zeisel et al., 2015; Olsson et al., 2016; Shekhar et al., 2016). For instance, Deng et al. (2014) showed that alleles were randomly expressed in mammalian cells; Buettner et al. (2015) discovered new cell subpopulations by exploring scRNA-seq data. The scRNA-seq technology has also been applied to the study of human pancreas (Table 1). Such as it performed transcriptome analysis of human pancreatic cells from healthy and type II diabetes (T2DM) donors (Li et al., 2016; Wang et al., 2016b; Lawlor et al., 2017a; Lawlor et al., 2017b). Xin et al. (2016) and Segerstolpe et al. (2016) illustrated the expression heterogeneity in human islet cells (e.g., apoptotic cells, mesenchymal cells, and α-cells). In addition, compared to healthy individuals, alterations in T2DM gene expression patterns and rich signaling pathways were also analyzed.

**TABLE 1:** Summary of scRNA-seq in pancreatic physiology and disease biology.

Events	Disease	Sample/Tissue	Technology	References
The development of pancreatic cells	NA	Normal pancreatic tissue	scRNA-seq	Gregg et al. (2012), Kimmel and Meyer (2010), Villani et al. (2019), Zeng et al. (2017), Sharon et al. (2019)
The heterogeneity of endocrine cells	NA	Normal pancreatic tissue	scRNA-seq	Dorrell et al. (2016), Fang et al. (2019), Gu et al. (2002), Grun et al. (2016), Qiu et al. (2017), Scheuner and Kaufman (2008)
The exploration of rare cells	NA	Normal pancreatic tissue	scRNA-seq	Cabrera et al. (2006), Muraro et al. (2016), Segerstolpe et al. (2016)
Changes of endocrine cells in diabetes	Diabetes	Pancreatic tissue in healthy adults and type 2 diabetic patients	scRNA-seq	Baron et al. (2016), Clark and Nilsson (2004), Dorajoo et al. (2017), Fang et al. (2019), Ma and Zheng (2018), Nomiyama et al. (2007)
			RePACT algorithm	
Neonatal diabetes	Diabetes	β-like cells differentiated *in vitro*	scRNA-seq	Balboa et al. (2018), Huopio et al. (2016)
The relationship between rare cells and diabetes	Diabetes	Pancreatic tissue in healthy adults and type 2 diabetic patients	scRNA-seq	Arrojo e Drigo et al. (2015), Ranjan et al. (2009), Unanue (2016)
New strategies for diabetes treatment	Diabetes	NA	scRNA-seq	Bruni et al. (2014), Millman et al. (2016), Pagliuca et al. (2014), Surguchov (2020)
The relationship between CAFs and PDAC	PDAC	PDAC cell lines	scRNA-seq	Amedei et al. (2014), Apte et al. (2013), Belle and Denardo (2019), Erkan et al. (2012), Patel et al. (2014)
		Mouse model	Single-cell proteomics	
		Human PDAC tissue		
New findings in pancreatic cancer metastasis	PDAC	PDAC patient blood	scRNA-seq	Ankeny et al. (2016), Clawson et al., 2017, Chaffer and Weinberg (2011)
		Mouse model		
The relationship between acinar metaplasia and PDAC	PDAC	Mouse model	scRNA-seq	Delgiorno et al. (2014), Li et al. (2016), Menheniott et al. (2016), Rotinen et al. (2018)
The relationship between genome changes and pancreatic cancer	Pancreatic cancer	Pancreatic tumor tissue	scRNA-seq	Biankin et al. (2012), Le et al. 2015, Waddell et al. (2015)

NA, not available.

**FIGURE 1:**
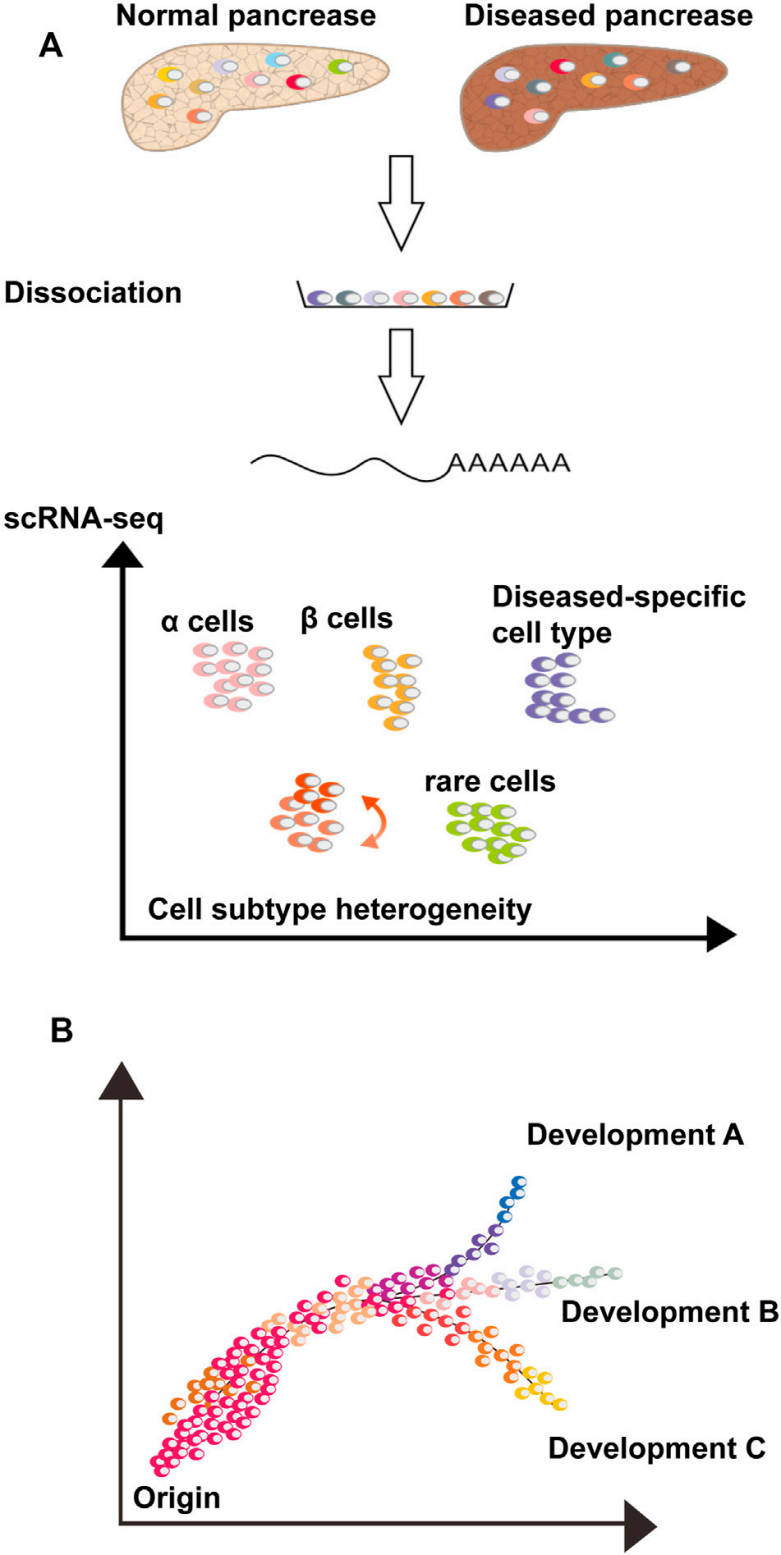
Single-cell RNA-sequencing analyses to study pancreaspathophysiology. **(A)** Dissociate normal and/or diseased pancreatic tissue into single cell suspension and scRNA-seq is perform. Thousands of transcripts of each cell are compressed in a 2D space, where each cell is a dot, and the distance between cells is a function of their similarity. Cells can be gathered into clusters or groups of clusters with different colors, possibly representing cell types or subtypes. The scRNA-seq allows the study of rare cell types, cell state and subtype heterogeneity, disease-specific cell types and cell-cell interactions via ligand-receptor analysis. **(B)** Computational analysis, such as pseudo-time diffusion mapping or RNA velocity, analyze pancreatic cell similarity and diversity, consent to track the differentiation process, clonal evolution and cell state transitions of a specific cell type or between different cell types.

**FIGURE 2:**
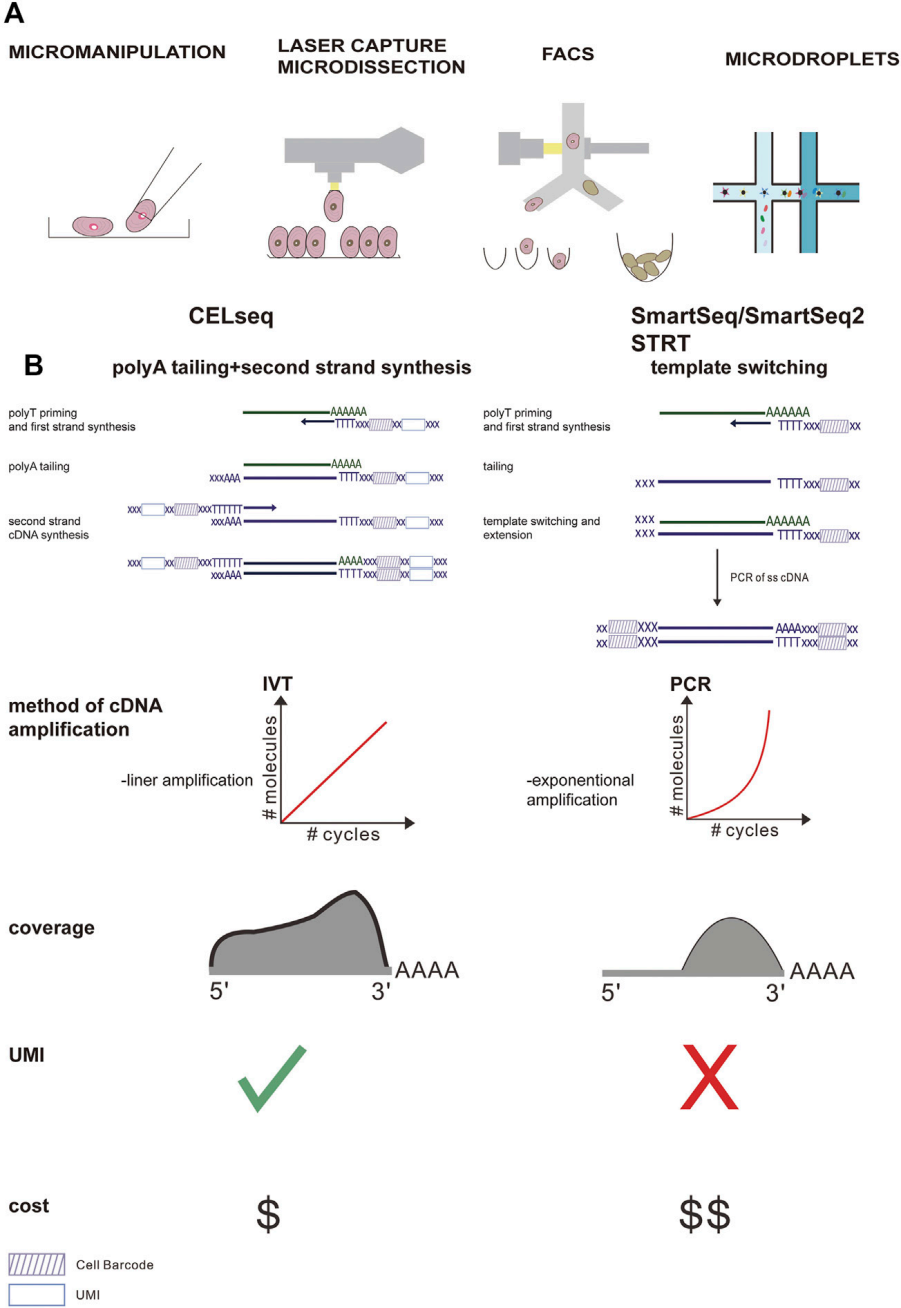
Comparison of the main steps of the scRNA-seq workflow and the most widely used protocol. **(A)** Different methods of single cell separation. **(B)** Smart-Seq uses polymerase chain reaction (PCR) for reverse transcription and cDNA amplification, while CEL-Seq uses *in vitro* transcription (IVT) for reverse transcription and cDNA amplification. In the CEL-Seq protocol, UMI and cell-specific barcodes are added during the reverse transcription process. In Smart-seq2, the gene coverage is full-length, while in CEL-Seq, only the 3′ part of the gene is sequenced. In addition, CEL-Seq reduces operating costs significantly compared to Smart-Seq.

In the scRNA-seq operation, the pancreatic tissue was first dissociated, and single cells were captured. Several workflow flowcharts for RNA sequencing are shown in Figures 1, 2. The dataset generated by scRNA-seq is large containing thousands of gene transcripts for each cell. These datasets are often displayed in a 2D space, where cells can be clustered according to similarity. In addition, single or multiple genes can be plotted on a separate t-SNE map (Li et al., 2017) (Figure 1A). Cell subpopulations, disease-specific cell types, rare cell populations, and intercellular interactions can be identified and studied using scRNA-seq. Furthermore, associated computer analysis, such as RNA velocity or pseudo-time-diffusion mapping (Figure 1B), allows tracing and examining of developmental pathways between cell types (Haghverdi et al., 2016; La Manno et al., 2018).

**FIGURE 3:**
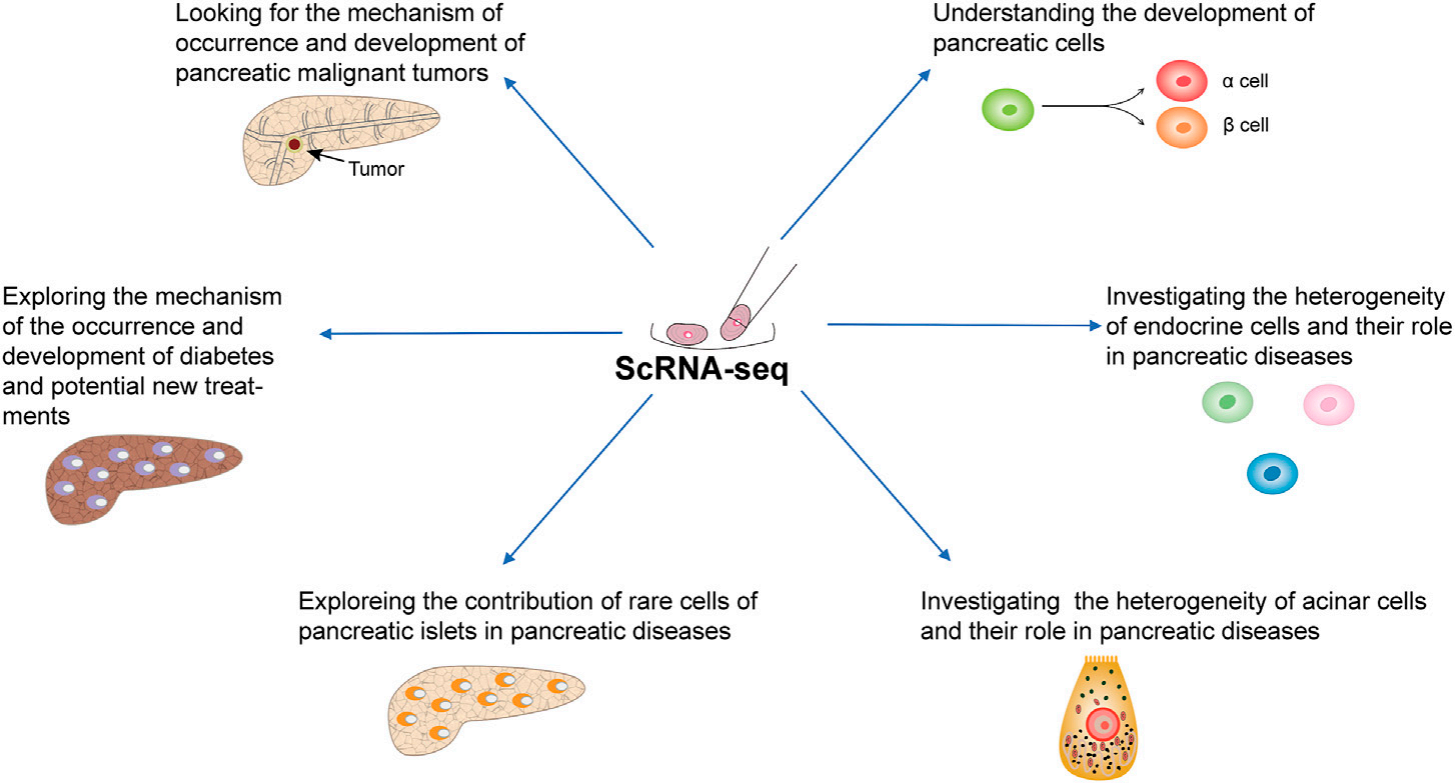
Application of scRNA-seq in the study of pancreatic physiology and pathology.

This review summarizes recent studies that used a single-cell approach to improve our understanding of the pancreas and pancreatic diseases (Figure 3).

## From Pancreatic Tissue to scRNA-Seq

The initial procedure of the scRNA-seq studies includes tissue lysis and single-cell separation, which can be acquired via different methods, including micromanipulation, fluorescence-activated cell sorting (FACS), laser capture microdissection (LCM), and microdroplet-based microfluidic technologies (Kolodziejczyk et al., 2015). Micromanipulation can be used to capture individual cells from samples containing a small number of cells. Although this method has a low throughput and is time-consuming, it can ensure that a single cell is obtained during each attempt (Grindberg et al., 2013; Xue et al., 2013; Yan et al., 2013). LCM capture of individual cells from tissue involves the use of a laser to attach individual cells from the tissue to a film, which can ultimately be removed (Frumkin et al., 2008). However, FACS remains one of the most widely applied techniques to select specific cell populations from tissues owing to its increased throughput. Microfluidic technologies based on high-throughput droplets, such as 10x chromium, are increasingly being used because of their high capture efficiency and low cost. Microfluidic technology first disperses single cells into water-in-oil droplets containing unique primers and barcode beads, and then uses continuous oil flow to separate them (Figure 2). The choice of single cell capture method depends to a large extent on the cell types of interest, cost, and number of cells in the tissue.

Following cell separation, the scRNA-seq library is produced by cell lysis, reverse transcribed into cDNA, the second-strand is synthesized, the cDNA is amplified using PCR or *in vitro* transcription (IVT), and ultimately deep sequencing takes place. These steps vary in different single-stranded nucleic acid sequence schemes (Figure 2). Briefly, methods with good technical support (such as smart-seq2) are more sensitive to gene detection, but the cost per cell is higher. Smart-seq2 can produce full-length transcripts and better detect low-redundant transcripts. Furthermore, it is suitable for discriminating fine distinctions in transcripts, which may be crucial for defining intermediate cell populations and developmental trajectories.

Although droplet-based cell separation methods have the remarkable advantage of being able to generate a large number of cells from specific tissues at a lower cost, it is associated with lower sensitivity. Hence, this method is suitable for obtaining rare cells and determining cell composition in tissues, although its accuracy in defining intermediate progenitor cells and developmental pathways may be affected (Yu and Xu, 2020).

## Application of scRNA-Seq in Pancreatic Physiology

### Growth and Development of the Pancreas

**FIGURE 4:**
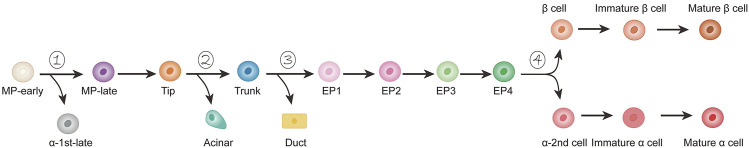
Developmental model of mouse pancreas α/β lineage. Starting from the early cells of MP, there are four branch nodes along the path of pancreatic development (1–4). Endocrine precursor (EP) cells can be divided into four stages (EP1–EP4).

The development of complex organs usually involves multiple procedures for cell fate selection (Yu et al., 2019). Since the pancreas is a unique organ in vertebrates and plays a crucial role in energy homeostasis by secreting metabolic hormones and digestive enzymes, a comprehensive understanding of the cell lineage differentiation pathways during the pancreas development, especially the regulation strategies of cell lineage separation points, is essential for stem cell differentiation into cell types required for regenerative medicine (Kimmel and Meyer, 2010). In recent years, scRNA-seq has been used to study endocrine and exocrine pancreatic lineages, as well as the developmental trajectories and regulatory strategies of intermediate progenitor cells along the developmental pathway. Yu et al. performed a scRNA-seq analysis on the purified pancreatic ancestry of diverse transgenic mouse strains from E9.5–E17.5, and defined four major branch lineage options along the pancreatic development (Figure 4). The first branching node was early multipotent progenitor (MP) cells, which moved along a branch into the late-stage MP cells and eventually differentiated into tip cells. Along the other branch, MP-early cells eventually differentiated into α-first cells. Tip cells represent the second node, which differentiated into acinar and trunk cells. Because trunk cells were heterogeneous, early trunk cells could be regarded as the third node, which branched into endocrine precursor (EP) cells and duct cells via the transient stages of trunk-EP and trunk-duct, respectively. Surprisingly, we found that EP cell differentiation involved four stages (EP1–EP4). EP4 cells were the fourth branch node from which islet lineage allocation was initiated (Yu et al., 2019). The identification of key lineage branch points is of great importance in understanding the regulatory logic and strategies used during cell fate selection in pancreatic lineage differentiation. Furthermore, it is important to study the dynamic changes in gene expression patterns in the process of cell fate selection and determine the cell population-specific transcription factors (TFs) as well as other genes related to the establishment of cell identity for further understanding of pancreatic development. Recently, Villani et al. (2019) identified a multipotent cluster of SOX9^+^/PTF1A^+^ cells using scRNA-seq and confirmed that human pancreatic SOX9^+^/PTF1A^+^ cells were uncommitted multipotent progenitor cells located at the tip of pancreatic epithelial cell expansion, directing self-renewal, and inducing pancreatic organ genesis.

### Growth and Development of α- and β-Cells

**FIGURE 5:**
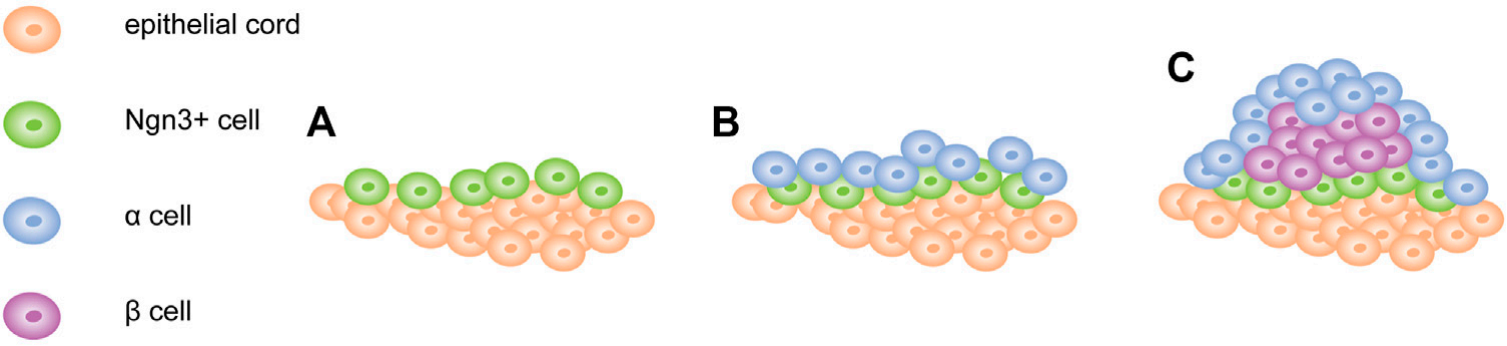
Model of pancreatic islet development. **(A)** Ngn3 + cells appear and remain attached to the surface of epithelial cord. **(B)** α cells first appear at the tip of the peninsula. **(C)** β cells form later and concentrate between α cells and cord. Newly formed endocrine cells continue to push out old cells and expand the peninsula.

Recent studies have found that pancreatic β-cell clumps for proper glycemic control are established in the early postnatal period, during which cell mass in humans was significantly increased due to an explosion of neonatal cell proliferation (Gregg et al., 2012). The outbreak was followed by a sharp decline in the proliferation of β-cells in the early postnatal period and a more gradual decline in the aging process. The ability of β-cells to proliferate decreases after birth; however, the intra- and extracellular signals that cause this decline remain unknown. To further explore this question, Zeng et al. (2017) used scRNA-seq to reconstruct the postnatal development trajectory of pancreatic β-cells. They separated β-cells at five different time points between birth and post-weaning, and then generated a single-cell transcriptome. Subsequently, they sorted all dissected β-cells based on transcriptional similarity, and developed an algorithm based on one-dimensional (1D) projections to construct a “pseudotemporal” trajectory of postpartum β-cell development. The algorithm proclaimed significant changes to have occurred in β-cell metabolism during the early postnatal period. Their results showed that the development of postpartum β-cells was associated with reduced mitochondrial reactive oxygen species (ROS) production and amino acid deprivation. Furthermore, they demonstrated that ROS and amino acids play a crucial role in the proliferation of postpartum β-cells. In addition, scRNA-seq was utilized to acquire a strict track of islet development. Based on this, Sharon et al. constructed a trajectory using a new algorithm to describe the transcriptional changes that occur with the endocrine progenitor cell islet formation. This figure shows that islet morphogenesis and endocrine cell differentiation are strongly related (Sharon et al., 2019). The combination of a transcription map and visual analysis of the developing pancreas provided a different explanation for the process of shaping the islets. Contrary to the mainstream view that islet formation does not involve the migration of multiple single cells, differentiated endocrine precursors maintain cell contact throughout the development process, and islets form an expanding peninsula (Sharon et al., 2019). Early differentiated α-cells appeared at the top of the peninsula, while later differentiated β-cells were formed at the rear. It laid the foundation for the final islet structure: the cells at the top of the peninsula were ultimately located at the periphery of the islets, while the β-cells that appeared at the bottom of the peninsula formed the core of the islets (Figure 5). Notably, human islets are composed of small core and shell components (Bonner-Weir et al., 2015). When several small peninsulas merged, it seemed to form a human structure. Through a single-cell spatiotemporal study of the developing pancreas, we concluded that ROS was an important driving force for β-cell proliferation and β-cell quality establishment in the early postpartum period, as well as the close connection between pancreatic islet morphology and endocrinology. Overall, this study provides a new framework for understanding the development of islets. Moreover, we are curious whether ROS drive the proliferation of β-cells during metabolic adaptation. Can a reasonable framework be constructed to induce islet formation *in vitro*?

### Heterogeneity of β-Cells

Glucose homeostasis is regulated by insulin and glucagon. Both α- and β-cells gathered in islets originate from Neurog3 (Ngn3^+^) progenitor cells during embryogenesis (Gu et al., 2002). These cells differentiate and mature functionally after birth (Qiu et al., 2017). For a long time, β-cells were thought to be functionally different, and the speed of insulin release and synthesis to differ between cells. The main regulator of insulin secretion is glucose, which stimulates insulin secretion at high levels and inhibits it at low levels. However, other stimulating factors, such as fatty acids, neuronal signals, amino acids, and hormones, also play a crucial role in determining the insulin secretion of the β-cell population. Are all β-cells equally responsive to all physiological signals? Are there any subpopulations that respond differently to specific signals? Increasing evidence (Roscioni et al., 2016) suggests that β-cells are heterogeneous. Recent studies have shown that β-cells can be divided into different subpopulations based on the differential expression of cell surface antigens or marker genes (Dorrell et al., 2016). Dorrell et al. (2016) and Bader et al. (2016) used scRNA-seq to identify a set of markers capable to distinguish β-cell subpopulations; however, in their studies, only two subpopulations were identified among the immature β-cell population. Further studies have shown that the two populations represented cells at different stages of maturity, and that the less mature cells might be generated by Ngn3^+^ endocrine progenitor cells. However, in adult β-cells, the differences between subpopulations were delicate and determined by only a few genes. In addition, the transcriptome profile of β-cells at the same maturity stage suggested homogeneity (Qiu et al., 2017). Therefore, we hypothesize that the heterogeneity of mature β-cells may be controlled at the post-transcriptional level, and that it can reflect different functional subpopulations.

**FIGURE 6:**
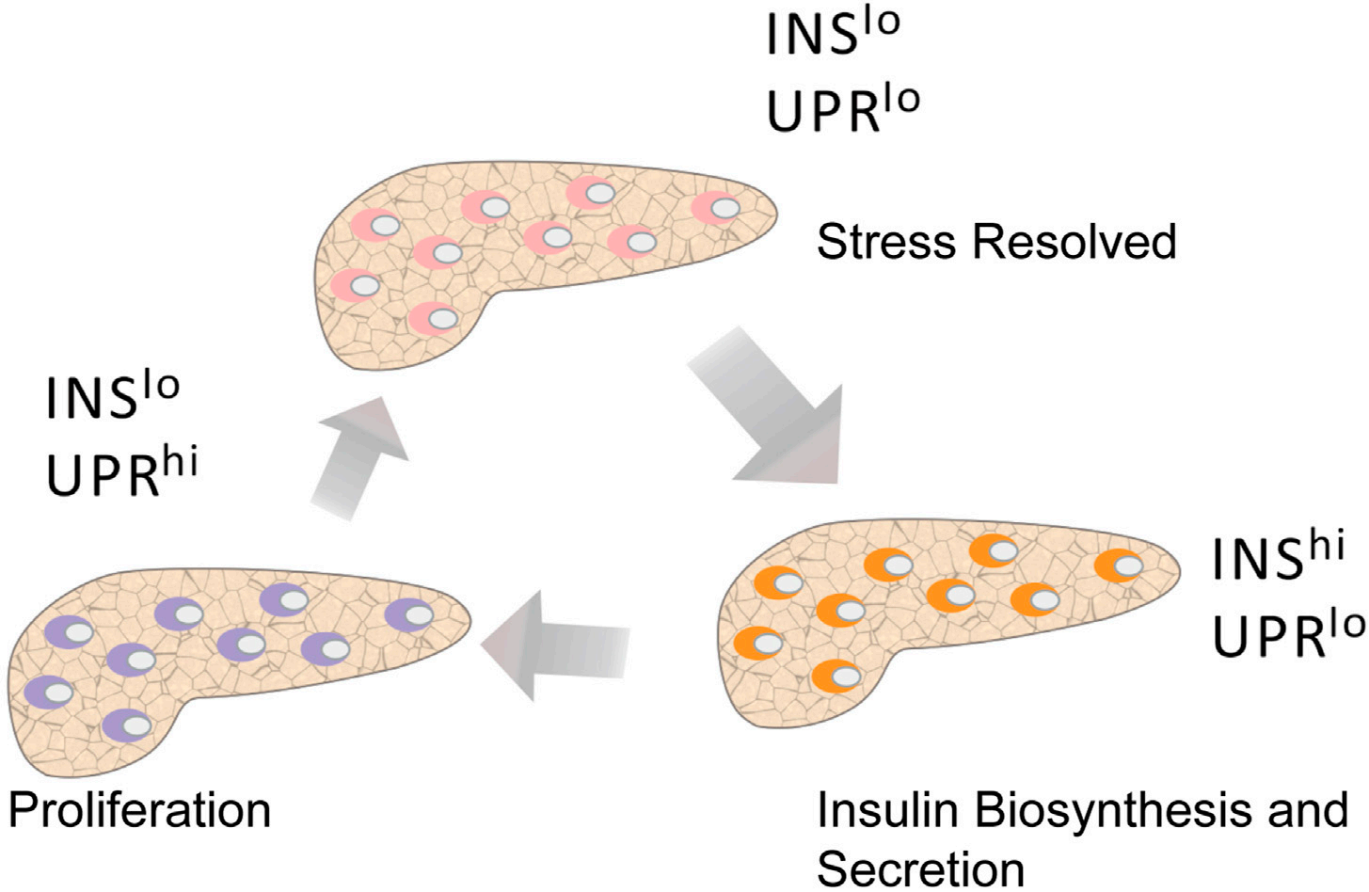
Pseudotime analysis shows the different states of β cells. Human β cells with active insulin biosynthesis and secretion (INS hi UPR lo) may become strained and transition to a recovery period (INS lo UPR hi) that includes UPR activation and low INS expression. After recovery, β-cells transition to a state characterized by low INS expression and low UPR activation (INS lo UPR lo), at which point they are almost ready to be actively secreted again. Among these states, the proliferating β-cells are mainly in a state of low INS expression and high UPR activation.

Recent analysis of RNA-seq data has revealed that human β-cell subpopulations may display a dynamic interchangeable state and the following states have been identified: 1) high unfolded protein response (UPR) and low insulin gene expression, 2) low UPR and low insulin gene expression, and 3) low UPR and high insulin gene expression (Xin et al., 2018) (Figure 6). In addition, the latest developments in scRNA-seq technology allowed unprecedented insights into gene expression patterns, which ultimately led to the identification of periods of high insulin biosynthesis activity associated with β cells and UPR activation periods associated with ER stress (Muraro et al., 2016; Fang et al., 2019; Wang and Kaestner, 2019). Under basal conditions, the biosynthesis of insulin accounted for more than 10% of the total protein production, while it was as high as 50% in the stimulated state (Scheuner and Kaufman, 2008). Proinsulin is a misfolding-prone protein, and 20% of synthetic proinsulin fails to reach its mature configuration (Liu et al., 2010; Simpson et al., 2011; Wang and Osei, 2011; Gorasia et al., 2016). If proinsulin misfolds, it can degrade or refold. However, during high insulin demand, misfolded proinsulin accumulates in the ER, leading to cell stress. To offset ER stress, the UPR mechanism is activated, protein folding activity is enhanced, ER workload is reduced, and the proportion of misfolded proteins is reduced (Fonseca et al., 2011). In this case, part of β-cells will be responsible for meeting the body’s demand of insulin, while the other part will be given time to recover from the pressure of its biosynthesis. Further analysis of β-cell heterogeneity showed that ER stress caused by UPR-mediated recovery and insulin production had a significant effect on human β-cells. We hypothesize that under pathological conditions the proportion of β-cells would change with disease progression. Long periods of high insulin demand during insulin resistance forces large numbers of β-cells to actively secrete insulin, thus reducing the recovery time from stress. Such continuous chronic cell stress increases the sensitivity to apoptotic factors. When the number of β-cells is greatly reduced and the necessary balance between activity and rest cannot be maintained, a decrease in insulin secretion is observed and T2DM develops (Dominguez-Gutierrez et al., 2019).

Traditionally, studies on the pancreas have evaluated β-cells and islets isolated from pancreatic tissue; however, their position in the pancreas or pancreatic islet components is rarely considered. Nevertheless, we found that in human type 1 diabetes, the loss of β-cells in the tail of the pancreas was more severe than that in the head, while for T2DM, most of the β-cell loss was observed in the head of the pancreas, indicating a functional differences between islets from different regions of the pancreas (Wang et al., 2013; Poudel et al., 2015).

In summary, β-cell heterogeneity highlights the potential for islet plasticity and how this islet functional change affects diabetes. However, to date, most studies examined dissociated cells, in which numerous transcriptome/protein characteristics may be altered. In addition, the molecular characteristics of subpopulations have also been well characterized, although little is known about their effects on the functional state and protein expression. The key to a comprehensive understanding of β-cell heterogeneity is the examination of a complete tissue, integrating multiple aspects of cell information, and establishing a model with better fidelity. This would provide a better understanding of the dynamic gene and protein expression. However, there are still some gaps in our knowledge: how can we define cell subpopulations in a robust way? What is the functional role and specific molecular basis of other endocrine cell heterogeneity (δ- and α-cells)? Further exploration of this topic might address these issues and potentially uncover new pathways and genes that can be exploited for improved treatment, induce β-cell regeneration, and discovery of novel technical information regarding islets and islet transplantation.

### Source of Acinar Cells

Most of the pancreas (95%) is composed of two types of exocrine cells: ductal and acinar cells. Acinar cells produce digestive enzymes, including lipase, amylase, and peptidase, while ductal cells secrete bicarbonate and transport digestive enzymes to the gastrointestinal tract (Steward et al., 2005; Whitcomb and Lowe, 2007). Although acinar cells account for most of the cells in the pancreas, the source of new acinar cells remains unclear. To explore this problem, Wollny et al. (2016) performed multi-chromatographic line tracking and scRNA-seq on acinar cells. They found progenitor-like acinar cells and confirmed that the acinar subpopulation was the source of new acinar cells in steady state, eventually producing terminally differentiation, post-mitotic binuclear acinar cells, which indicates the clonal heterogeneity among acinar cells (Wollny et al., 2016). Further study of acinar cells at the single-cell level will reveal the hierarchical relationship between acinar cell types, which will undoubtedly be of great significance for understanding the adult pancreas.

### Exploration of Rare Cells

Among the five cell types of pancreatic islets, the most common are α- and β-cells, which make up approximately 75–85% of pancreatic islets (Cabrera et al., 2006). The other cell types are represented by δ-cells, ε-cells and pancreatic polypeptide cells (γ-cells, which are refered to PP cells), which mainly secrete the pancreatic polypeptides ghrelin and somatostatin, respectively. Transcriptome-wide studies are usually performed on islets, which masks the contribution of rare cell types (Muraro et al., 2016). The scRNA-seq technology facilitates the research and analysis of rare cell types in pancreatic islets (Li et al., 2016). Segerstolpe et al. (2016) examined the rare cell types of pancreatic islets and found that δ-cells express leptin and ghrelin receptors, while ε-cells express diverse receptor molecules for endorphins, glycoproteins, and neurotransmitters. In brief, these results indicate that δ- and ε-cells could act as pancreatic islet sensors. In addition, MHC class II molecules were found to be expressed in acinus cells, suggesting that pancreatic exocrine cells such as antigen-presenting cells associated with immune interfaces may influence the pathogenesis of type I diabetes mellitus (T1DM).

## Application of Single Cell Sequencing in diabetes

Diabetes can be classified into T1DM and T2DM, which represents the seventh leading cause of death in the United States of America (Menke et al., 2015). T1DM is caused by the destruction of more than 80% of β-cells and mediated by autoimmunity in the pancreas, while T2DM is caused by the enhancement of insulin resistance, which eventually aggravates the effects of dysfunctional β-cells, rendering the remaining β-cells insufficient to meet the homeostatic needs (Halban et al., 2014; Johnson, 2016). Therefore, replacing the missing or abnormal β-cells remains the most important goal in diabetes research. However, with the development of scRNA-seq technology, it has been shown that the occurrence and development of diabetes is not only related to β-cells, but also α-cells and rare cells. Next, we will expand on the occurrence and progression of diabetes and summarize the potential new treatment methods.

### β-Cell Associated Diabetes

T2DM is characterized by impaired insulin secretion and insulin resistance. The insufficient insulin secretion is associated with the dysfunction or loss of β-cells and the development of insulin resistance, which refers to a decrease in insulin sensitivity of insulin-targeted tissues or cells (Ma and Zheng, 2018). Here, we focused on the relationship between β-cells and diabetes.

Studies have shown that β-cell dysfunction is closely related to obesity and T2DM. Nevertheless, since T2DM and obesity are two highly correlated diseases, it is challenging to demarcate the differences and similarities between them. Traditional transcriptome analysis requires a large number of patients with pure endocrine cell subpopulations to reach statistical significance, which is difficult to obtain in practice. The scRNA-seq technology allows transcriptome analysis in a single cell and further deepens the analysis of complex or rare tissues, including islets (Baron et al., 2016; Dorajoo et al., 2017). Fang et al. (2019) developed a RePACT algorithm based on scRNA-seq data. This is a universal single-cell algorithm that can identify relevant disease genes with high sensitivity, identify different cell changes in T2DM as well as obesity, and further determine numerous specific and common genes from β-cells. They confirmed 1,188 obesity locus genes and 1,368 T2DM locus genes associated with β-cells (Fang et al., 2019). Obesity is known to increase the risk of developing diabetes. As hypothesized, there are various genetic commonalities between these two diseases. For instance, IAPP, SPP1, and GAPDH were downregulated in both obese and T2DM patients. GAPDH is an important enzyme involved in glucose metabolism and glycolysis. Both SPP1 and IAPP are generally regarded as risk factors for diabetes and obesity. In particular, IAPP forms amyloid, which was related to T2DM-specific cytotoxicity (Clark and Nilsson, 2004). Alternatively, SPP1 mediates the infiltration of obesity-induced macrophages into adipose tissue and insulin resistance (Nomiyama et al., 2007).

**FIGURE 7:**
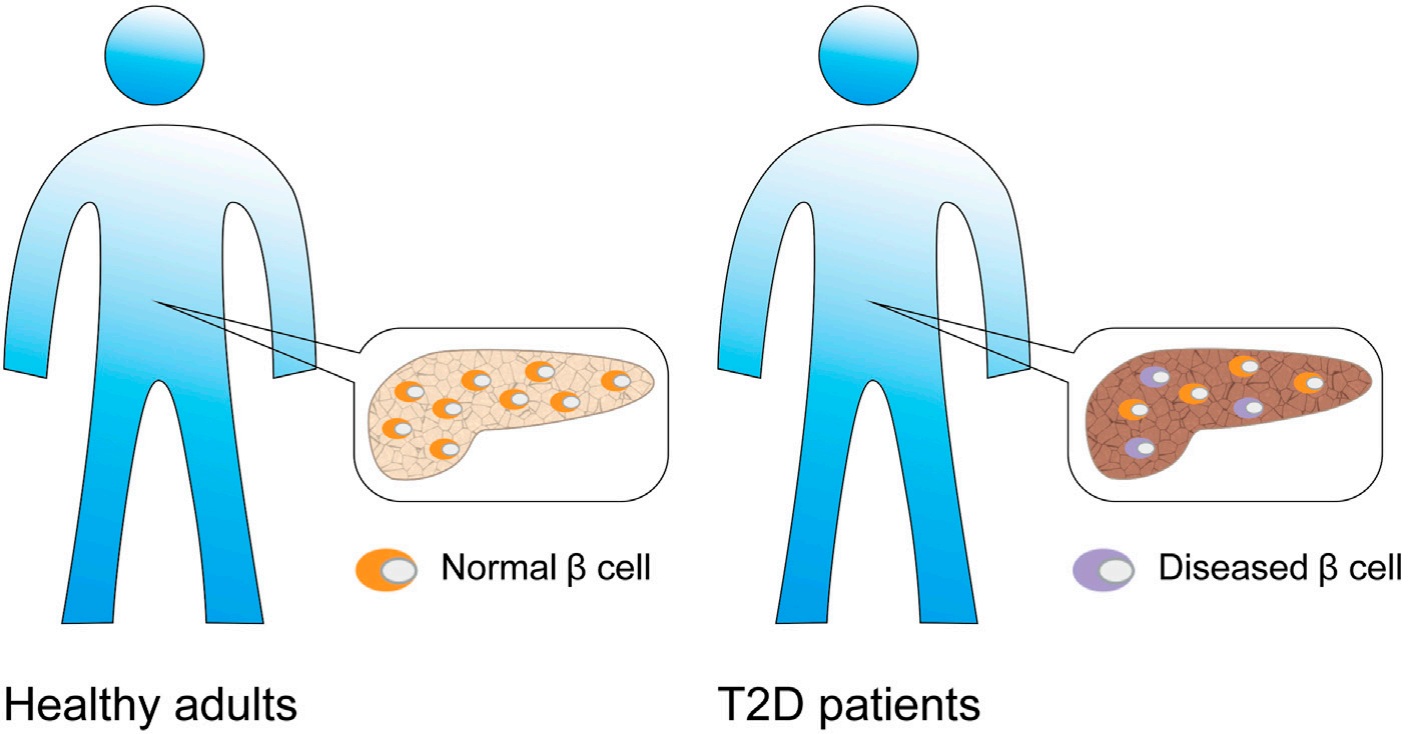
β-cell associated diabetes. Compared with normal adults, patients with type 2 diabetes have β cell dysfunction, and the number of β cells is also significantly reduced.

According to relevant reports, the β cell function of numerous patients declined even before they were clearly diagnosed with T2DM (Ma and Zheng, 2018). Additionally, other studies confirmed that the number of β-cells in diabetic patients decreases by 20–65% (Rahier et al., 2008; Meier and Bonadonna, 2013) (Figure 7). Kahn (2003) studied the effects of β-cell dysfunction and insulin resistance on the pathophysiology of T2DM. Yoon et al. (2003) surveyed the number of β-cells in T2D patients. Although these studies were aimed at studying the functional and number abnormalities of β-cells in T2DM, they were mainly based on a large number of cell analyses and provided only average information about cell populations. To reveal the underlying mechanism of β-cell dysfunctions in T2DM, Ma and Zheng (2018) utilized scRNA-seq to analyze the β-cells of T2DM donors. They found that the β-cells of T2DM donors can normally express INS under low cell stress conditions; however, they exhibit abnormal functionality under high stress conditions. Healthy β-cells can maintain INS expression at normal levels and handle high cellular pressures. Furthermore, a correlation analysis indicated that oxidative stress might represent a crucial factor affecting INS expression in T2DM patients. BAX-, CAPN1-, CAPN2-, and TNFR1-dependent pathways might represent effective therapeutic targets for the treatment of β-cell apoptosis in T2DM. Caspase and INS are also differentially expressed donors compared to healthy controls (Ma and Zheng, 2018).

### Neonatal Diabetes

Insulin gene mutations are a major cause of neonatal diabetes. Neonatal diabetes usually occurs in the first 6 months of life and causes the cessation of insulin production. Babies with early diabetes usually exhibit mutations in the gene encoding insulin. This implies that they can still synthesize some insulin but are not sufficient to maintain blood sugar stability, which ultimately leads to a gradual stop of insulin production within a few months. It is believed that this is because β-cells are affected by the toxic effects of mutant insulin. The structure of insulin is influenced by the insulin gene mutation. Consequently, insulin accumulates in the β-cells, generating high cellular stress and finally causing cell death (Balboa et al., 2018). However, the underlying mechanism remains unknown.

Finnish people are known to carry the C96R (the same cysteine as the Akita C96Y mutation) and C109Y insulin mutations. Therefore, to further study the mechanism underlying neonatal diabetes occurrence and development, Balboa et al. (2018) extracted human induced pluripotent cells (iPSCs) from Finnish donors, differentiated them into β-like cells *in vitro*, and performed scRNA-seq analysis (Huopio et al., 2016; Balboa et al., 2018). Their results indicated that misfolded proinsulin in combination with INS triggered the induction of ER stress, which affects the growth of INS mutant β-cells by influencing their proliferation but not apoptosis. *In vitro* and *in vivo* experiments showed that reduced proliferation led to a lower percentage of INS^+^ cells in INS mutant cells. Increased ER stress also led to decreased mTORC1 signaling and mitochondrial changes, which are essential for the proliferation and function of β-cells. Importantly, their results suggested that the INS mutation that caused neonatal diabetes was pathogenic in the development of the pancreas because of the failure of neonatal β-cell expansion. Theoretically, a new perspective for future treatments of mutant insulin-related diabetes might be achieved by briefly stimulating mTORC1, given that the stimulation is performed during the neonatal period (Balboa et al., 2018).

In summary, these findings may be related to the development of T2DM later in life.

### Dedifferentiation of α- and β-Cells in Diabetes

Recent studies have shown that α- and β-cells dedifferentiate during the development of diabetes. To detect the state of rare cells and reveal the population heterogeneity of cells, Wang et al. (2016b) utilized scRNA-seq to investigate islets from multiple deceased organ donors, including healthy adults, patients with T1DM or T2DM, and children. According to the relevant literature, the arrangement and types of endocrine cells in islets vary greatly among different mammalian species (Steiner et al., 2010). For example, a rodent islet core contains as many as 90% insulin-producing β-cells, while human islets show more heterogeneous endocrine cells with only approximately 54% β-cells (Brissova et al., 2005). Through scRNA-seq, the transcriptome of human pancreatic endocrine cells has been determined and four different developmental and physiological states were defined: infancy, normal adulthood, T1D, and T2D. They found that the transcription status of α- and β-cells was not fixed in infancy, but matured with age. In addition, under diabetic condition, α- and β-cells showed more immature genetic characteristics, suggesting the dedifferentiation process (Wang et al., 2016b).

One particularly striking result of their study was that both α- and β-cell transcriptomics from T2DM donors exhibited the expression profile characteristics of their juvenile counterparts, which indicated that some of them had returned to an immature state. Additionally, analysis of animal models showed that the pancreatic islets of old mice had enhanced insulin secretion compared to those of young pancreatic islets. Therefore, the similarity between young α- cells and β-cells and T2DM indicated that T2DM might be partly due to a compensatory mechanism associated with the failure to synthesize insulin, and often with age (Avrahami et al., 2015).

### Relationship Between Rare Cells and Diabetes

Although islet separation is a common operation, the close connection of all these auxiliary and endocrine cells in islets complicates the separation and purification processes of homogenous populations. We generally attribute changes in gene and protein expression in intact islets to β-cells; however, this obviously ignores the heterogeneous population of endocrine cells, as well as macrophages, endothelial cells, fibroblasts, and glial cells which together constitute islets (Ranjan et al., 2009; Arrojo e Drigo et al., 2015; Unanue, 2016). In diabetic patients, it has been shown that corresponding transcriptional and functional changes also occur in α-cells, rare cells, and the vascular system (Dai et al., 2013; Brissova et al., 2018; Lam et al., 2018). Unfortunately, it is difficult to distinguish the changes in β-cells in intact islets from other changes.

**FIGURE 8:**
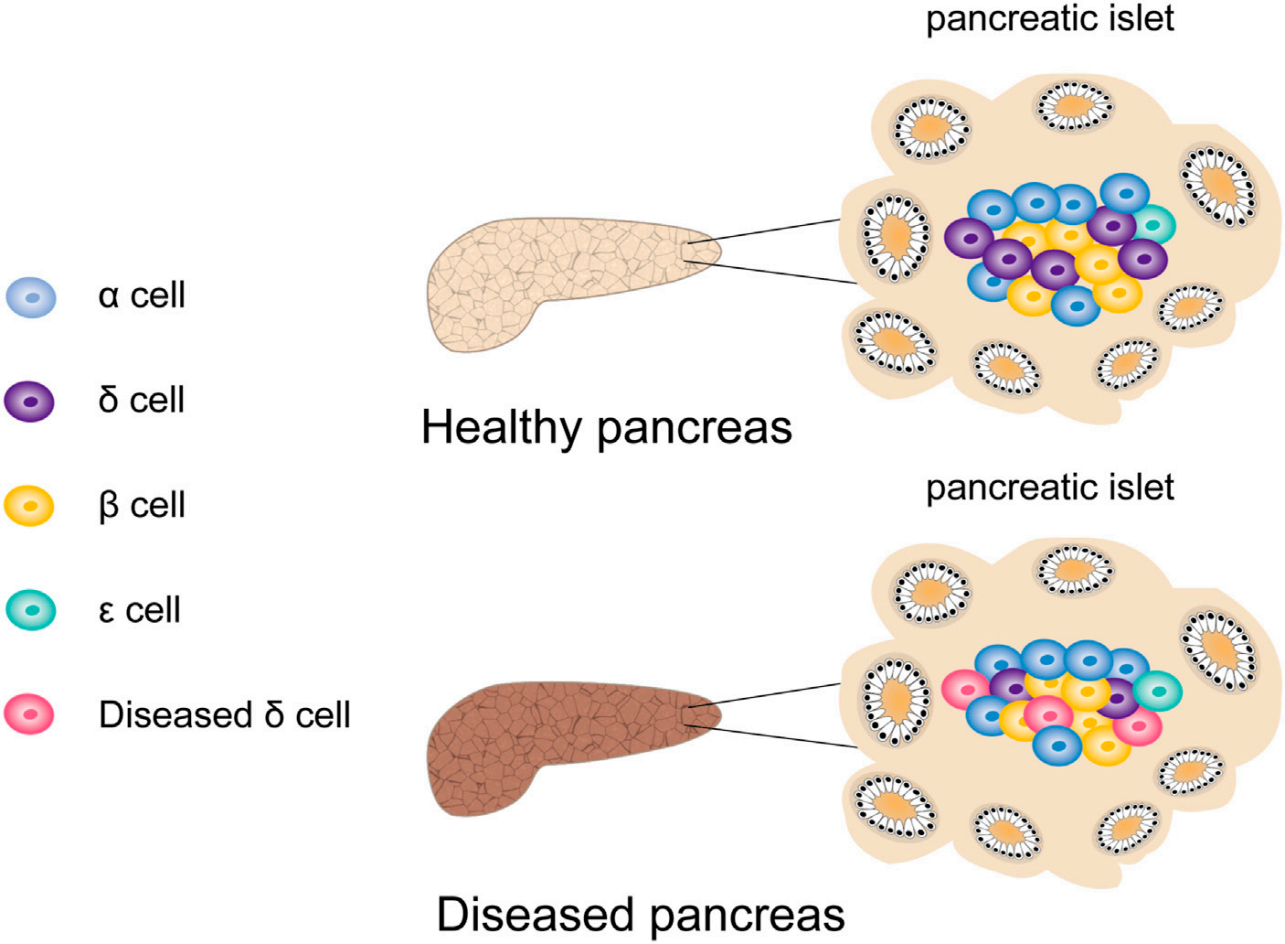
The effect of rare cells on the development of diabetes. In addition to β cell dysfunction in patients with type 2 diabetes, the dysfunction of rare cells such as δ cells is also a potential factor for the development of diabetes.

Along with the scRNA-seq development, high-throughput analysis of transcriptomes across cell types, subpopulations, and states has become feasible (Sandberg, 2014). Segerstolpe et al. (2016) performed scRNA-seq on thousands of human pancreatic islet cells from T2DM patients and healthy donors. They found that although the number of ε- and δ-cells in pancreatic islets is small, their transcriptome suggested that they might have a previously unrecognized crucial role in pancreatic islet homeostasis. Through a variety of receptors, δ-cells can sense and respond to various hormone signals. Moreover, they expressed GHRL receptors, which allowed them to accept input from ε-cells or to release GHRL during digestion. The transcriptome of δ-cells indicates the expression of leptin receptors. In addition, Lawlor et al. showed that in addition to β-cell dysfunction, δ-cell dysfunction was also a potential factor in the development of monogenic diabetes (Lawlor et al., 2017a) (Figure 8). However, there are still various unresolved issues regarding the role of rare cells in pancreatic islets, which urgently require further research.

### New Strategies for Treating Diabetes

Diabetes is characterized by causing chronic hyperglycemia and systemic metabolic complications, and can cause multiple organ damage in the long term (Surguchov, 2020). Although the incidence rate of diabetes is increasing, the disease remains incurable, and our understanding of the potential pathogenesis remains insufficient. Although the current treatments can successfully alleviate the symptoms, they cannot prevent the occurrence of long-term complications and require patients to engage in life-long therapy. Hence, great efforts have been made in the field of diabetes research to develop new therapeutic strategies for the regeneration of β-cells and prevention of disease progression.

**FIGURE 9:**
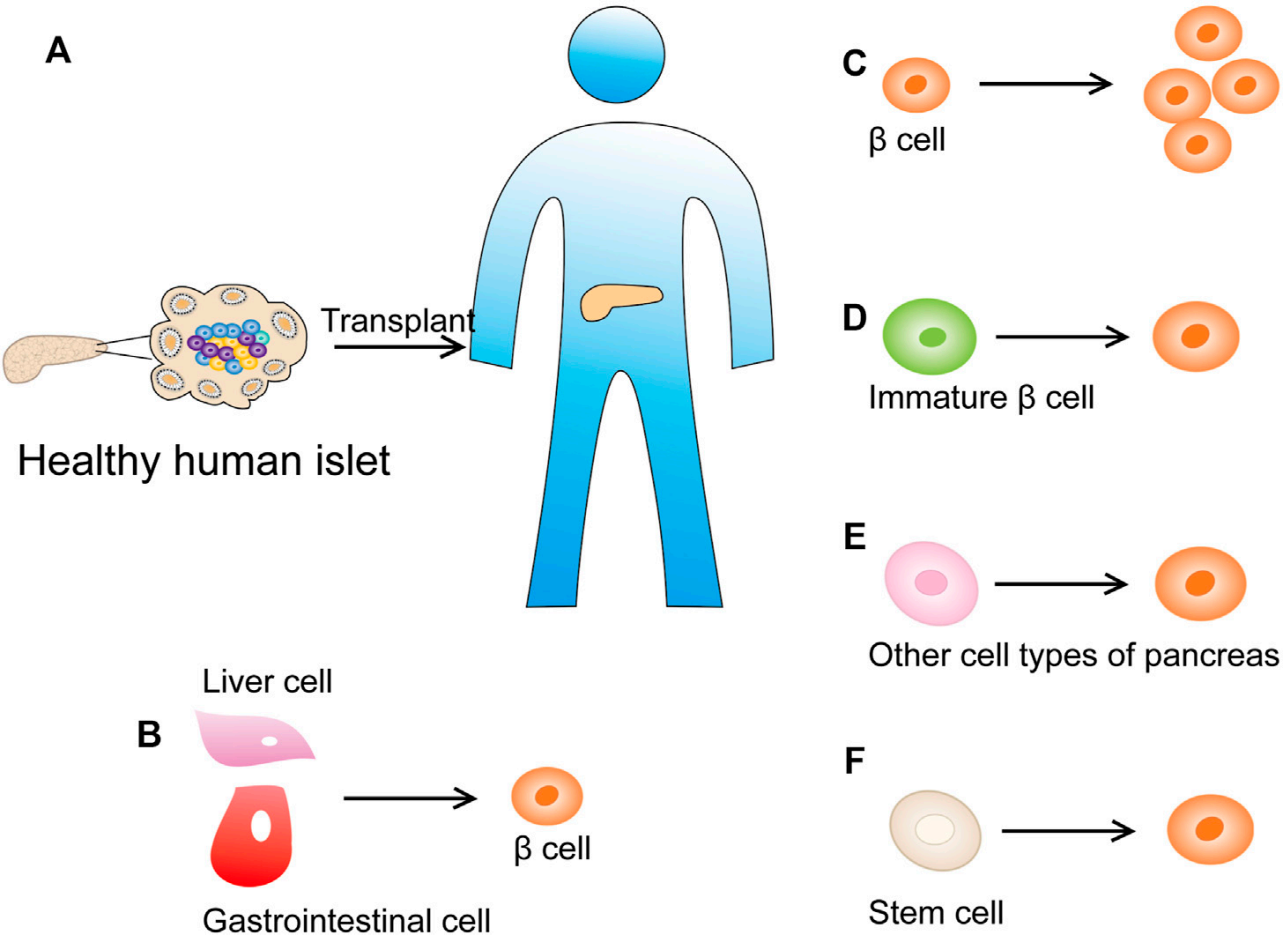
New strategies for diabetes treatment. **(A)** Human islet transplantation has successfully become a treatment method for diabetic patients. **(B)** Inducing cells in the adult pancreas or other metabolically active organs (such as the intestine or liver) into β cells that can secrete insulin. **(C)** Promote the replication of the remaining β cells of the pancreatic islets. **(D)** Promote the maturation of immature β-cell subpopulations. **(E)** Reprogram other cell types into insulin-producing β-like cells (such as pancreatic acinar cell and α cells). **(F)** Differentiate β cells from human stem cells *in vitro*.

Human islet transplantation represents a successful treatment option for T1DM patients (Figure 9A). These patients do not respond to conventional and intensive insulin therapy and exhibit complications with renal failure (Bruni et al., 2014). Nevertheless, due to the shortage of donors and the risks related to lifelong immunosuppression, new alternative therapies need to be developed. Currently, two main treatments are studied to restore dysfunctional β-cells: 1) endogenous β-cell regeneration and 2) *in vitro* stem cell differentiation into β-cells (Figure 9F). The latter provides great hope for cell replacement therapy. In the past few years, the differentiation of human embryonic stem cells from patient-derived induced pluripotent stem cells into β-like cells that respond to glucose has been achieved (Pagliuca et al., 2014; Millman et al., 2016). Most importantly, these cells were shown to secrete insulin in diabetic mice and restore normal blood sugar levels (Russ et al., 2015). However, the differentiation efficiency and function of β-like cells generated *in vitro* need to be further improved before they can be used in humans. Therefore, a more comprehensive understanding of the postpartum β-cell maturation process and the identification of biomarkers associated with β-cells at different maturation stages will greatly promote the development of this field. Furthermore, their safety must be ensured, because immature stem cells may have the potential to cause teratomas.

Inducing β-cell regeneration from cells in the pancreas or other metabolic organs (such as the intestine or liver) is an attractive method to overcome the aforementioned obstacles (Figure 9B). The main ways to restore the quality of functional β-cells *in situ* include promoting the maturation of immature β-cell subpopulations (Figure 9D), accelerating the replication of remaining β-cells (Figure 9C), and mobilizing other cell types to reprogram into insulin-producing β-like cells [pancreatic acinar cells showed plasticity *in vitro* and *in vivo*, and can be used as a source of β-cell regeneration (Migliorini et al., 2014)] (Figure 9E).

Although single-cell genomics in the human pancreas is still in its early phase, it has provided a novel insights into the transcriptional program of pancreatic endocrine cells. The expression of diabetes risk genes and leptin receptors in δ-cells strongly suggests that δ-cells play a crucial role in maintaining glucose balance, although they were present in relatively small numbers (Baron et al., 2016).

The most promising method to restore β-cell quality is the induction of β-cell maturation and/or replication. The scRNA-seq data from postnatal rat cells further illustrated the immune cell characteristics and suggested that ER stress, ROS, MAPK, SRF, TGF, PDGF, and WNT signaling regulate post-natal cell proliferation and maturation (Qiu et al., 2017; Zeng et al., 2017). Surprisingly, PDGF and MAPK signal transduction as well as ER stress were also associated with potential adult β-cell subpopulations (Baron et al., 2016; Muraro et al., 2016; Wang et al., 2016a). Therefore, regulating the above pathways might represent a method to reactivate the expansion and maturation of healthy β-cells in diabetic patients.

Reprogramming cells from islets into β-cells is another method of β-cell regeneration. Previous studies suggested that the transformation of α-cells into β-cells in mice contributes to a steady-state β-cell renewal (Grun et al., 2016; van der Meulen et al., 2017). Since severe β-cell exhaustion can trigger the differentiation of α-cells into β-cells in mice, and α-cells are the most proliferative type of endocrine cells, their use might be a promising way to promote β-cell regeneration (Thorel et al., 2010; Wang et al., 2016a) (Figure 9E). In this regard, it is crucial to identify the transformation signals that drive α-cell proliferation and the transformation of α-cells into β-cells during homeostasis and injury. Using scRNA-seq technology, the adult proliferative α-cells have been analyzed and SHH signaling has been confirmed as a candidate pathway for regulating their proliferation (Wang et al., 2016a; Wang et al., 2016b; Segerstolpe et al., 2016).

Single-cell research on pancreatic endocrine cells allowed for the identification of several pathways and genes that were essential to drive the maturation and proliferation of β-cells, elucidated the heterogeneity of endocrine cells, illuminated some of their potential underlying mechanisms, and provided novel therapeutic targets and subpopulations that can be used for β-cell regeneration.

## Application of Single-Cell Sequencing in Pancreatic Cancer

Pancreatic ductal adenocarcinoma (PDAC) is one of the most common cancers worldwide, with a high mortality rate, a 5-year survival rate of 10%, and little improvement in the last decade. It is projected to become the second leading cause of cancer-related deaths by 2030 (Clawson et al., 2017; Schlesinger et al., 2020). Because PDAC lacks early symptoms, cannot be surgically removed, and there is no effective alternative treatment, the available treatments are only a short delay in disease progression. PDAC is usually diagnosed late and most patients will die within 1 year following diagnosis (Clawson et al., 2017; Chan-Seng-Yue et al., 2020). Therefore, it is paramount to investigate the development of pancreatic cancer from early lesions to late-stage cancers. Further we will discuss the progress made in single-cell pancreatic cancer imaging.

### Role of Cancer-Associated Fibroblasts in PDAC

In recent years, an increasing number of studies have shown that CAFs are associated with the tumor development, including tumor growth and metastasis, angiogenesis, immunosuppression, and fibrosis (Clawson et al., 2017). Nevertheless, in the context of cancer, the exact function of CAFs is still under debate, and this debate is most relevant to PDAC, which is characterized by tumor-related malignant hyperplasia (Belle and Denardo, 2019).

The stroma of PDAC is composed of a complex ecosystem of endothelial cells, CAFs, and immune cells that provide the basis for tumor cells to regulate tumor growth and invasion (Erkan et al., 2012; Apte et al., 2013; Amedei et al., 2014; Patel et al., 2014). CAF accounts for most of the tumor stroma, and depletion of a specific subpopulation of CAFs slows PDAC progression and improves anti-tumor immunity in some PDAC models (Hamada et al., 2012; Ene-Obong et al., 2013; Feig et al., 2013; Ligorio et al., 2019). However, studies in some mouse models have shown that depleting CAFs leads to more aggressive PDAC behavior (Ozdemir et al., 2014; Rhim et al., 2014). Thus, it has been suggested that the relative change in CAF content could have different effects on tumor progression (Moffitt et al., 2015).

In addition, the combination of protein analysis platforms and scRNA-seq provided new solutions to deciphering the relationship between transcriptional programs (epithelial-to-mesenchymal transformation; EMT and proliferation; PRO) and signaling pathways (STAT3 and MAPK) in the model system and individual cancer cells of primary human tumors. For instance, TGFB1 secreted by CAFs is believed to be a promoter of DP (PRO^+^EMT, double-positive) phenotypes in various PDAC cell lines, and TGFB1, which has been proven to be directly involved in MAPK signaling in pancreatic carcinoma, hepatocellular carcinoma, head and neck squamous cell carcinoma, as well as lung carcinoma (Liu et al., 2014; Principe et al., 2017; Tang et al., 2017; Wang et al., 2018). The combined effect of STAT3 and MEK inhibitors in KRAS-mutant pancreatic cancer and colon cancer has been confirmed (Zhao et al., 2015). Ligorio et al. (2019) showed the effects of dual activation of STAT3 and MAPK on the phenotypes of PDAC cells and the relationship between this signal transduction and TGFB1 produced by CAFs. These results highlight the significance of the changes in stromal CAF composition and the sensitivity of drug combinations.

Furthermore, Biffi et al. (2019) used scRNA-seq to detect CAF heterogeneity in PDAC tissues from mouse models. Based on previous studies, they found that CAF could express two molecular subpopulations, myofibroblast CAFs (myCAFs) and inflammatory CAF (iCAFs) (Ohlund et al., 2017). Furthermore, IL1/JAK/STAT3 and TGFβ/Smad3 signaling in mouse tumors were shown using scRNA-seq to be key regulatory pathways controlling the heterogeneity and function of MyCAFs and iCAFs (Biffi et al., 2019). Considering that there might be other endocrine subpopulations within the PDAC stromal microenvironment, scRNA-seq has been used to investigate the different CAF subpopulations. A map of cluster enrichment was drawn by analyzing the sequence data of the individual tumor cells. Notably, new CAF populations have been discovered in both mouse models and human PDAC tissues. This new CAF subpopulation has specific molecular markers consistent with MHC II and can induce CD69 and CD25 activation in T cells. Therefore, these antigen-presenting cells were identified as antigen presenting CAFs (APCAFs) (Elyada et al., 2019).

**FIGURE 10:**
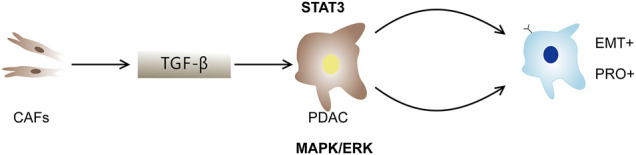
The role of CAFs in PDAC. CAFs interact with PDAC cells to induce PDAC cells to transform into PRO, EMT and DP subpopulations. TGF-β, STAT3 and MAPK/ERK signal pathways promote the development of DP population.

This study describes different types of PDAC cells from circulating tumor cells and solid tumors. PRO phenotypes, EMT phenotypes, and co-expression subpopulations were found in PDAC cell populations (Ting et al., 2014). At the same time, CAFs can also inhibit, reverse, or promote cancer cells through a complex mechanism (Gore and Korc, 2014; Laklai et al., 2016). Luo et al. used single-cell proteomics and scRNA-seq to further investigate how CAFs affect PDAC primary cancer cell mutations (Luo et al., 2020). Ligorio et al. (2019) demonstrated that CAFs could induce PDAC cells to transform into PRO, DP, and EMT subpopulations through interaction with PDAC cells. MAPK/ERK, STAT3, and TGFβ signaling pathways have been shown to be involved in the formation of PDAC subtypes (Figure 10). Most importantly, they found that the development of these specific PDAC cell phenotypes was categorically influenced by the co-culture mode of different proportions of CAFs and tumor cells. Additionally, the spatial structure of tumors and the composition of cell types have been shown to exhibit different effects on the growth and progression of tumors (Ligorio et al., 2019).

### New Discovery of Single Cell Sequencing in the Metastasis of Pancreatic Cancer

**FIGURE 11:**
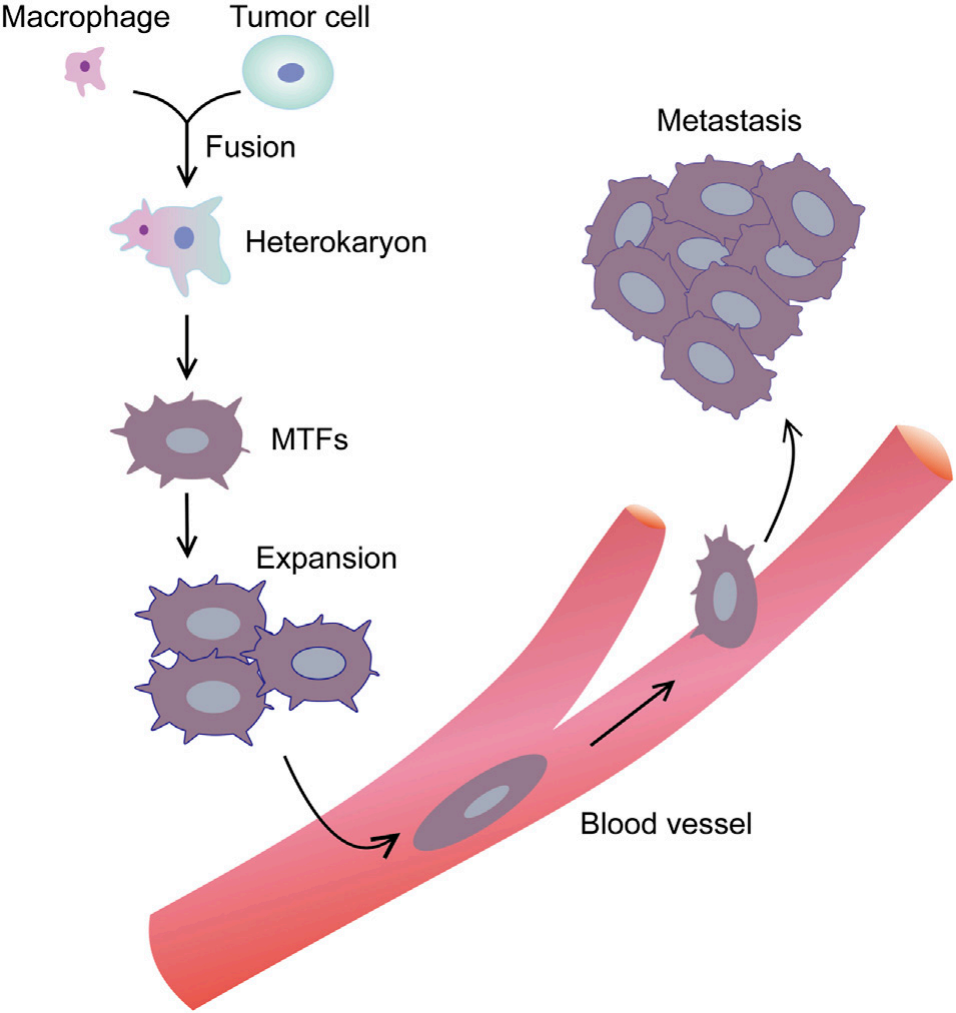
Macrophage-tumor cell fusion hypothesis. Macrophage is attracted near tumor cell. The 2 cells begin to fuse with each other, and through sequencing, recombination and/or reprogramming of genetic material, tumor cell with a highly aggressive macrophage phenotype can be generated.

Similar to most cancers, PDAC mortality is also caused by metastatic dissemination. At all stages of PDAC, the proportion of circulating tumor cells (CTCs) is high (Kulemann et al., 2015; Ankeny et al., 2016). Although there are various models of so-called metastatic cascades, the characteristics of CTCs producing metastatic lesions remain unclear (Clawson et al., 2017). Which CTCs can induce metastasis-initiating cells (MICs), and how MICs can discover suitable locations for the growth of metastatic lesions (Chaffer and Weinberg, 2011)? As for the former, one inference is that EMT-altered tumor cells around the primary cancer promote the release of tumor stem cells, and these cells may represent MICs (Chaffer and Weinberg, 2011; Karamitopoulou, 2012; Karamitopoulou et al., 2013). Nevertheless, this inference does not solve the problem of how MICs discover a suitable location to establish metastasis and proliferate (Clawson, 2013). Another metastasis theory involves the fusion of macrophages with tumor cells (MTFs) (Dittmar et al., 2013; Lazova et al., 2013). Tumor cells with a highly aggressive macrophage phenotype can be generated by sequencing, recombining, and/or reprogramming of the genetic material (Powell et al., 2011) (Figure 11). This view is supported by recent reports from human cancers, as well as a large amount of support from animal models (Lazova et al., 2013). Nevertheless, the mechanism of this phenomenon is unclear, which seems to be inconsistent with the EMT/stem cell hypothesis (Clawson, 2013).

Clawson et al. (2017) reported that similar MTFs cultured from the blood of PDAC patients showed similar expression of macrophage and epithelial/pancreatic/stem cell marker combinations. At the same time, scRNA-seq analysis showed that LINE-1 retrotransposons and diversified metastasis-related markers (especially CXCR4/CD44/CD74/MIF signal axis) were expressed at high levels. In related experiments, when cultured PDAC MTFs were transplanted into the pancreas of mice *in situ*, they formed well differentiated “islands,” while not forming visible tumors in other faraway places. Nevertheless, they have been detected to spread widely to various tissues, including the lung, spleen, liver, and submucosa. They were discovered as small clusters of cells or single-celled, usually irregular and large. There was no significant increase in the number of cells in multiple tissues between 4 and 12 weeks, although metastatic cells were detected in the lungs at 12 weeks. MTFs also altered the expression of some markers following propagation (Clawson et al., 2017). We speculate that the expression of MIF within distant tissues via scattered MTFs may promote the colonization of metastatic cells.

In addition to CTCs, cancer stem cells (CSCs) have been shown to play a crucial role in the treatment of drug resistance and malignant tumor recurrence (Court et al., 2015; Lapin et al., 2017; Agnoletto et al., 2019; Lytle et al., 2019). Lytle et al. (2019) used scRNA-seq to determine the molecular expression profiles of pancreatic CSCs (Luo et al., 2020). The MSI^+^ cell map constructed by Lytle and Ferguson defines the characteristics of CSCs and is consistent with the expression levels of Csf1r, IL34, and IL10Rβ in stromal cells. These factors may be controlled by retinoic acid receptor-related orphan receptor gamma (RORγ). The scRNA-seq revealed that RORγ expression was significantly upregulated in pancreatic CSCs, and was determined to be a prognostic marker as well as a potential therapeutic target for pancreatic carcinoma. Due to the unique molecular characteristics and small number of CSCs, the scRNA-seq technique was highly beneficial to uncovering the transcriptome of CSCs and providing new insights into the metastasis of PDAC and the treatment of drug resistance (Luo et al., 2020).

### Role of Acinar Metaplasia in Pancreatic Cancer

Acinar metaplasia is the first step in pancreatic carcinoma. The scRNA-seq of mouse pancreases from the pre-invasion phase until tumor formation has provided new insights into PDAC. However, an overview of the events underlying the change of acinar cells from normal to metaplasia and finally to malignant cells, which is of high important for understanding the development of PDAC, is missing (Schlesinger et al., 2020).

Dissecting the occurrence of PDAC has a huge potential to improve early screening, which is challenging in pancreatic carcinoma. Although acinar metaplasia is a crucial early step in PDAC development, our understanding of the molecular identity of the cells involved in this process and its contribution to malignant tumors is incomplete. A previous study used scRNA-seq on samples collected from a genetically engineered mouse model and determined the uniquely expressed genes in metaplastic cells in the diseased pancreas. Foxq1 and Onecut2 are two of the main TFs identified to be expressed in advanced metaplastic epithelial cells originating from acinar cells; however, they were not expressed in any early metaplastic cells or other cells in the pancreatic tissue. Onecut2 is a member of the CUT homeodomain TF family and is expressed in metaplasmic cells of mouse and human cancer cells. In addition, Onecut2 has recently been shown to play a key role in prostate carcinoma, although its function in PDAC has not been determined (Rotinen et al., 2018). According to previous analyses, Onecut2 might regulate the expression of genes that control Kras downstream signal transduction. Furthermore, high Onecut2 expression was associated with poor prognosis, and Onecut2 might represent an underlying driving force for the development of early PDAC.

Foxq1 was shown to be expressed in several tumor types and may facilitate tumor initiation, proliferation, metastasis, and invasion; however, its function in PDAC remains unclear (Li et al., 2016). Similar to Onecut2, Foxq1 is expressed in mouse PDAC and KRT19^+^ humans cells, emphasizing its underlying importance. Although a few genes, such as Sox9, Onecut2, Foxq1, and Krt19 are expressed in all metaplastic cluster cells, we detected metaplastic cells containing several subpopulations with different transcriptional programs. By using large-scale scRNA-seq at different time points, we can analyze the early and late stages of metaplastic cells, accurately confirm their various identities, identify previously unanalyzed metaplasia types that are localized on pancreatic intraductular epithelial tumors (PANINs), and explore their development over time.

In addition, Schlesinger et al. (2020) further analyzed the cellular heterogeneity of vesicle cells identifying six vesicle metaplasia cell types and states, including stomach-specific cell types. The early metaplastic cells and acinar cells exhibited continuous changes in one of the two fates: alternative gastricization or tumorigenic. Therefore, gastric metaplasia acinar cells may not have the potential for malignant development. Moreover, GKN1, GKN2, and TFF1 expressed in stomach pit-like cells were thought to be gastric tumor suppressor genes, and since mouse TFF1 knockout could induce gastric cancer, these findings support the anti-tumor effect of these genes (Menheniott et al., 2016). Previous studies have shown that the number of metaplastic tubule-like cells decreases as the infiltrating pre-lesion transforms into a tumor (Delgiorno et al., 2014).

In summary, we show that precancerous lesions include more than one meta oncogenic cell type, and that the meta oncogenic cells can interact with each other and with other cell types in the tissue based on their expression profiles.

### Genomic Changes in Pancreatic Cancer

According to the relevant statistics, most pancreatic cancer patients have an effective response time from a few months to a year, while others have no effective response and show tumor growth in first-line chemotherapy (Le et al., 2015; Waddell et al., 2015). Patients with resectable tumors (stage I or II) might progress to stage IV within a few months, while others relapse several years later, and a few patients are cured. However, genomic research continues to support the view that the disease is homogeneous, with four genes changing repeatedly: CDKN2A, TP53, SMAD4, and KRAS (Biankin et al., 2012; Witkiewicz et al., 2015). It is not clear why clinical heterogeneity occurs if most tumors grow through the same mutation pathway. Therefore, Michelle et al. studied in detail the relationship between the mutation process related to genome instability and the clinical heterogeneity of pancreatic cancer (Chan-Seng-Yue et al., 2020). The scRNA-seq and genomic analyses revealed that there is heterogeneity between the basal-like and classical-like pancreatic cancer subtypes. There were at least two different molecular subclusters for each subtype. Basal-like tumors were more aggressive and existed in two states known as Basal-like A and B. The squamous expression pattern is the main factor that distinguishes the two basal phenotypes. Clinical studies have shown that Basal-like-A tumors accumulate in metastatic diseases, while Basal-like-B accumulate in resectable diseases, which supports the conclusion that the squamous expression programs were selected for disease progression. According to results obtained through scRNA-seq, most tumors have both classical and basal-like tumor cells, but the scale of these cells is distinct. A transcript continuum at the RNA-seq level has been created, and the mixed tumors represented the main problem. In addition, we found that copy number events in the genome were associated with basal-like phenotypes to a certain extent. Both the clinical stage and severity of the imbalance were associated with this relationship. Most metastatic tumors had a certain degree of KRAS mutation imbalance, and we hypothesize that this phenomenon is associated to the high frequency of metastatic genome doubling. Finally, our research revealed that the heterogeneity of the disease was caused by genome instability (Chan-Seng-Yue et al., 2020).

## Conclusion

The scRNA-seq is a revolutionary technology, its advantage lies in the successful use of unprecedented high resolution to study the biology of diabetes and pancreatic tumors. This technology showed the heterogeneity of rare pancreatic cell types and cell populations, as well as characterize the interactions between them. Although sRNA-seq and the related computer analyses have transformed the study of complex cells as well as tissues and brought great hope in the field of pancreatic development, there are still issues that need to be addressed. First, scRNA-seq has high operational requirements. For instance, dissociation is a key step in sRNA-seq technology. The degree of dissociation can directly cause changes in the transcriptome. Therefore, further optimization should be conducted to achieve a greater dissociation yield without biasing the end results. In addition, scRNA-seq is expensive, data analysis is time-consuming, and requires skilled bioinformatic support. scRNA-seq has revolutionized the understanding of pancreatic endocrine function, the biology of diabetes and cancer, and has allowed the detailed examination of tumor microenvironments. The current challenge is to integrate the newly acquired knowledge and translate it into effective new therapies that can alleviate the clinical burdens associated with pancreatic related diseases. However, throughout the review, we further developed our understanding of diabetes and its treatments. Based on existing studies, we identified the mechanisms that induce the proliferation and maturation of β-cells and elucidated the feasibility of endocrine cell *trans*-differentiation. Furthermore, we emphasized the importance of changes in the stromal CAF composition during tumor therapy and provided a new therapeutic target for PDAC metastasis and drug resistance. In conclusion, we believe that these novel treatments have great developmental prospects and can produce unprecedented curative effects on diseases.

## Abbreviations

APCAF, antigen presenting CAF; CAF, cancer-associated fibroblasts; CTC, circulating tumor cell; CSC, cancer stem cells; cDNA, complementary DNA; EP cell, endocrine precursors cell; iCAF, inflammatory CAF; EMT, epithelial-mesenchymal transition; FACS, fluorescence-activated cell sorting; hi, high; IL, interleukin; INS, insulin; iPSC, induced pluripotent stem cell; IVT, *in vitro* transcription; LCM, laser capture microdissection; lo, low; MP cell, multipotent progenitor cell; myCAF, Myofibroblast CAF; PanIN, pancreatic intraepithelial neoplasia; PCR, polymerase chain reaction; PDAC, pancreatic ductal adenocarcinoma; PRO, proliferative; ScRNA-seq, single-cell RNA sequencing; TME, tumor microenvironment; TFs, transcription factors; UPR, unfolded protein response; UMI, unique molecular identifier.

## References

[B1] Agnoletto C. Corrà F. Minotti L. Baldassari F. Crudele F. Cook W. (2019). Heterogeneity in Circulating Tumor Cells: The Relevance of the Stem-Cell Subset. Cancers 11, 483–519. 10.3390/cancers11040483 PMC6521045 30959764

[B2] Amedei A. Niccolai E. Prisco D. (2014). Pancreatic Cancer: Role of the Immune System in Cancer Progression and Vaccine-Based Immunotherapy. Hum. Vaccin. Immunother. 10, 3354–3368. 10.4161/hv.34392 25483688 PMC4514060

[B3] Ankeny J. S. Court C. M. Hou S. Li Q. Song M. Wu D. (2016). Circulating Tumour Cells as a Biomarker for Diagnosis and Staging in Pancreatic Cancer. Br. J. Cancer 114, 1367–1375. 10.1038/bjc.2016.121 27300108 PMC4984454

[B4] Apte M. V. Wilson J. S. Lugea A. Pandol S. J. (2013). A Starring Role for Stellate Cells in the Pancreatic Cancer Microenvironment. Gastroenterology 144, 1210–1219. 10.1053/j.gastro.2012.11.037 23622130 PMC3729446

[B5] Arrojo e Drigo R. Ali Y. Diez J. Srinivasan D. K. Berggren P.-O. Boehm B. O. (2015). New Insights into the Architecture of the Islet of Langerhans: a Focused Cross-Species Assessment. Diabetologia 58, 2218–2228. 10.1007/s00125-015-3699-0 26215305

[B6] Avrahami D. Li C. Zhang J. Schug J. Avrahami R. Rao S. (2015). Aging-Dependent Demethylation of Regulatory Elements Correlates with Chromatin State and Improved β Cell Function. Cel Metab. 22, 619–632. 10.1016/j.cmet.2015.07.025 PMC4598285 26321660

[B138] Bader E. Migliorini A. Gegg M. Moruzzi N. Gerdes J. Sara S. S. (2016). Identification of Proliferative and Mature β-Cells in the Islets of Langerhans. Nature 535, 430–434. 10.1038/nature18624 27398620

[B7] Balboa D. Saarimäki-Vire J. Borshagovski D. Survila M. Lindholm P. Galli E. (2018). Insulin Mutations Impair Beta-Cell Development in a Patient-Derived iPSC Model of Neonatal Diabetes. Elife 7. 10.7554/eLife.38519 PMC6294552 30412052

[B8] Baron M. Veres A. Wolock S. L. Faust A. L. Gaujoux R. Vetere A. (2016). A Single-Cell Transcriptomic Map of the Human and Mouse Pancreas Reveals Inter- and Intra-cell Population Structure. Cel Syst. 3, 346–360 e344. 10.1016/j.cels.2016.08.011 PMC5228327 27667365

[B9] Belle J. I. Denardo D. G. (2019). A Single-Cell Window into Pancreas Cancer Fibroblast Heterogeneity. Cancer Discov. 9, 1001–1002. 10.1158/2159-8290.CD-19-0576 31371323

[B10] Biankin A. V. Waddell N. Kassahn K. S. Gingras M. C. Muthuswamy L. B. Johns A. L. (2012). Pancreatic Cancer Genomes Reveal Aberrations in Axon Guidance Pathway Genes. Nature 491, 399–405. 10.1038/nature11547 23103869 PMC3530898

[B11] Biffi G. Oni T. E. Spielman B. Hao Y. Elyada E. Park Y. (2019). IL1-Induced JAK/STAT Signaling Is Antagonized by TGFβ to Shape CAF Heterogeneity in Pancreatic Ductal Adenocarcinoma. Cancer Discov. 9, 282–301. 10.1158/2159-8290.CD-18-0710 30366930 PMC6368881

[B12] Bonner-Weir S. Sullivan B. A. Weir G. C. (2015). Human Islet Morphology Revisited. J. Histochem. Cytochem. 63, 604–612. 10.1369/0022155415570969 25604813 PMC4530393

[B13] Brissova M. Fowler M. J. Nicholson W. E. Chu A. Hirshberg B. Harlan D. M. (2005). Assessment of Human Pancreatic Islet Architecture and Composition by Laser Scanning Confocal Microscopy. J. Histochem. Cytochem. 53, 1087–1097. 10.1369/jhc.5C6684.2005 15923354

[B14] Brissova M. Haliyur R. Saunders D. Shrestha S. Dai C. Blodgett D. M. (2018). α Cell Function and Gene Expression Are Compromised in Type 1 Diabetes. Cel Rep. 22, 2667–2676. 10.1016/j.celrep.2018.02.032 PMC6368357 29514095

[B15] Buettner F. Natarajan K. N. Casale F. P. Proserpio V. Scialdone A. Theis F. J. (2015). Computational Analysis of Cell-To-Cell Heterogeneity in Single-Cell RNA-Sequencing Data Reveals Hidden Subpopulations of Cells. Nat. Biotechnol. 33, 155–160. 10.1038/nbt.3102 25599176

[B16] Cabrera O. Berman D. M. Kenyon N. S. Ricordi C. Berggren P.-O. Caicedo A. (2006). The Unique Cytoarchitecture of Human Pancreatic Islets Has Implications for Islet Cell Function. Pnas 103, 2334–2339. 10.1073/pnas.0510790103 16461897 PMC1413730

[B17] Chaffer C. L. Weinberg R. A. (2011). A Perspective on Cancer Cell Metastasis. Science 331, 1559–1564. 10.1126/science.1203543 21436443

[B18] Chan-Seng-Yue M. Kim J. C. Wilson G. W. Ng K. Figueroa E. F. O’Kane G. M. (2020). Transcription Phenotypes of Pancreatic Cancer Are Driven by Genomic Events during Tumor Evolution. Nat. Genet. 52, 231–240. 10.1038/s41588-019-0566-9 31932696

[B19] Clark A. Nilsson M. R. (2004). Islet Amyloid: a Complication of Islet Dysfunction or an Aetiological Factor in Type 2 Diabetes? Diabetologia 47, 157–169. 10.1007/s00125-003-1304-4 14722650

[B20] Clawson G. A. (2013). Fusion for Moving. Science 342, 699–700. 10.1126/science.1244270 24202164

[B21] Clawson G. A. Matters G. L. Xin P. Mcgovern C. Wafula E. Depamphilis C. (2017). "Stealth Dissemination" of Macrophage-Tumor Cell Fusions Cultured from Blood of Patients with Pancreatic Ductal Adenocarcinoma. PLoS One 12, e0184451. 10.1371/journal.pone.0184451 28957348 PMC5619717

[B22] Court C. M. Ankeny J. S. Hou S. Tseng H.-R. Tomlinson J. S. (2015). Improving Pancreatic Cancer Diagnosis Using Circulating Tumor Cells: Prospects for Staging and Single-Cell Analysis. Expert Rev. Mol. Diagn. 15, 1491–1504. 10.1586/14737159.2015.1091311 26390158 PMC4893319

[B23] Dai C. Brissova M. Reinert R. B. Nyman L. Liu E. H. Thompson C. (2013). Pancreatic Islet Vasculature Adapts to Insulin Resistance through Dilation and Not Angiogenesis. Diabetes 62, 4144–4153. 10.2337/db12-1657 23630302 PMC3837044

[B24] Delgiorno K. E. Hall J. C. Takeuchi K. K. Pan F. C. Halbrook C. J. Washington M. K. (2014). Identification and Manipulation of Biliary Metaplasia in Pancreatic Tumors. Gastroenterology 146, 233–244 e235. 10.1053/j.gastro.2013.08.053 23999170 PMC3870045

[B25] Deng Q. Ramsköld D. Reinius B. Sandberg R. (2014). Single-cell RNA-Seq Reveals Dynamic, Random Monoallelic Gene Expression in Mammalian Cells. Science 343, 193–196. 10.1126/science.1245316 24408435

[B26] Dittmar T. Nagler C. Niggemann B. Zanker K. S. (2013). The Dark Side of Stem Cells: Triggering Cancer Progression by Cell Fusion. Cmm 13, 735–750. 10.2174/1566524011313050005 23642055

[B27] Dominguez-Gutierrez G. Xin Y. Gromada J. (2019). Heterogeneity of Human Pancreatic β-cells. Mol. Metab. 27, S7–S14. 10.1016/j.molmet.2019.06.015 PMC6768494 31500834

[B28] Dorajoo R. Ali Y. Tay V. S. Y. Kang J. Samydurai S. Liu J. (2017). Single-cell Transcriptomics of East-Asian Pancreatic Islets Cells. Sci. Rep. 7, 5024. 10.1038/s41598-017-05266-4 28694456 PMC5504042

[B29] Dorrell C. Schug J. Canaday P. S. Russ H. A. Tarlow B. D. Grompe M. T. (2016). Human Islets Contain Four Distinct Subtypes of β Cells. Nat. Commun. 7, 11756. 10.1038/ncomms11756 27399229 PMC4942571

[B30] Elyada E. Bolisetty M. Laise P. Flynn W. F. Courtois E. T. Burkhart R. A. (2019). Cross-Species Single-Cell Analysis of Pancreatic Ductal Adenocarcinoma Reveals Antigen-Presenting Cancer-Associated Fibroblasts. Cancer Discov. 9, 1102–1123. 10.1158/2159-8290.CD-19-0094 31197017 PMC6727976

[B31] Ene–Obong A. Clear A. J. Watt J. Wang J. Fatah R. Riches J. C. (2013). Activated Pancreatic Stellate Cells Sequester CD8+ T Cells to Reduce Their Infiltration of the Juxtatumoral Compartment of Pancreatic Ductal Adenocarcinoma. Gastroenterology 145, 1121–1132. 10.1053/j.gastro.2013.07.025 23891972 PMC3896919

[B32] Erkan M. Hausmann S. Michalski C. W. Fingerle A. A. Dobritz M. Kleeff J. (2012). The Role of Stroma in Pancreatic Cancer: Diagnostic and Therapeutic Implications. Nat. Rev. Gastroenterol. Hepatol. 9, 454–467. 10.1038/nrgastro.2012.115 22710569

[B33] Fang Z. Weng C. Li H. Tao R. Mai W. Liu X. (2019). Single-Cell Heterogeneity Analysis and CRISPR Screen Identify Key β-Cell-Specific Disease Genes. Cel. Rep. 26, 3132–3144 e3137. 10.1016/j.celrep.2019.02.043 PMC6573026 30865899

[B34] Feig C. Jones J. O. Kraman M. Wells R. J. B. Deonarine A. Chan D. S. (2013). Targeting CXCL12 from FAP-Expressing Carcinoma-Associated Fibroblasts Synergizes with Anti-PD-L1 Immunotherapy in Pancreatic Cancer. Proc. Natl. Acad. Sci. 110, 20212–20217. 10.1073/pnas.1320318110 24277834 PMC3864274

[B35] Fonseca S. G. Gromada J. Urano F. (2011). Endoplasmic Reticulum Stress and Pancreatic β-cell Death. Trends Endocrinol. Metab. 22, 266–274. 10.1016/j.tem.2011.02.008 21458293 PMC3130122

[B36] Frumkin D. Wasserstrom A. Itzkovitz S. Harmelin A. Rechavi G. Shapiro E. (2008). Amplification of Multiple Genomic Loci from Single Cells Isolated by Laser Micro-dissection of Tissues. BMC Biotechnol. 8, 17. 10.1186/1472-6750-8-17 18284708 PMC2266725

[B37] Gorasia D. G. Dudek N. L. Safavi-Hemami H. Perez R. A. Schittenhelm R. B. Saunders P. M. (2016). A Prominent Role of PDIA6 in Processing of Misfolded Proinsulin. Biochim. Biophys. Acta (Bba) - Proteins Proteomics 1864, 715–723. 10.1016/j.bbapap.2016.03.002 26947243

[B38] Gore J. Korc M. (2014). Pancreatic Cancer Stroma: Friend or Foe? Cancer Cell 25, 711–712. 10.1016/j.ccr.2014.05.026 24937454 PMC4821630

[B39] Gregg B. E. Moore P. C. Demozay D. Hall B. A. Li M. Husain A. (2012). Formation of a Human β-Cell Population within Pancreatic Islets Is Set Early in Life. J. Clin. Endocrinol. Metab. 97, 3197–3206. 10.1210/jc.2012-1206 22745242 PMC3431572

[B40] Grindberg R. V. Yee-Greenbaum J. L. Mcconnell M. J. Novotny M. O'shaughnessy A. L. Lambert G. M. (2013). RNA-sequencing from Single Nuclei. Proc. Natl. Acad. Sci. 110, 19802–19807. 10.1073/pnas.1319700110 24248345 PMC3856806

[B41] Grün D. Muraro M. J. Boisset J.-C. Wiebrands K. Lyubimova A. Dharmadhikari G. (2016). De Novo Prediction of Stem Cell Identity Using Single-Cell Transcriptome Data. Cell Stem Cell 19, 266–277. 10.1016/j.stem.2016.05.010 27345837 PMC4985539

[B42] Gu G. Dubauskaite J. Melton D. A. (2002). Direct Evidence for the Pancreatic Lineage: NGN3+ Cells Are Islet Progenitors and Are Distinct from Duct Progenitors. Development 129, 2447–2457. 10.1242/dev.129.10.2447 11973276

[B43] Haghverdi L. Büttner M. Wolf F. A. Buettner F. Theis F. J. (2016). Diffusion Pseudotime Robustly Reconstructs Lineage Branching. Nat. Methods 13, 845–848. 10.1038/nmeth.3971 27571553

[B44] Halban P. A. Polonsky K. S. Bowden D. W. Hawkins M. A. Ling C. Mather K. J. (2014). β-Cell Failure in Type 2 Diabetes: Postulated Mechanisms and Prospects for Prevention and Treatment. Dia Care 37, 1751–1758. 10.2337/dc14-0396 PMC4179518 24812433

[B45] Hamada S. Masamune A. Takikawa T. Suzuki N. Kikuta K. Hirota M. (2012). Pancreatic Stellate Cells Enhance Stem Cell-like Phenotypes in Pancreatic Cancer Cells. Biochem. Biophysical Res. Commun. 421, 349–354. 10.1016/j.bbrc.2012.04.014 22510406

[B46] Huopio H. Miettinen P. J. Ilonen J. Nykänen P. Veijola R. Keskinen P. (2016). Clinical, Genetic, and Biochemical Characteristics of Early-Onset Diabetes in the Finnish Population. J. Clin. Endocrinol. Metab. 101, 3018–3026. 10.1210/jc.2015-4296 27167055

[B47] Janiszewska M. Liu L. Almendro V. Kuang Y. Paweletz C. Sakr R. A. (2015). *In Situ* single-cell Analysis Identifies Heterogeneity for PIK3CA Mutation and HER2 Amplification in HER2-Positive Breast Cancer. Nat. Genet. 47, 1212–1219. 10.1038/ng.3391 26301495 PMC4589505

[B48] Johnson J. D. (2016). The Quest to Make Fully Functional Human Pancreatic Beta Cells from Embryonic Stem Cells: Climbing a Mountain in the Clouds. Diabetologia 59, 2047–2057. 10.1007/s00125-016-4059-4 27473069

[B49] Kahn S. E. (2003). The Relative Contributions of Insulin Resistance and Beta-Cell Dysfunction to the Pathophysiology of Type 2 Diabetes. Diabetologia 46, 3–19. 10.1007/s00125-002-1009-0 12637977

[B50] Karamitopoulou E. (2012). Tumor Budding Cells, Cancer Stem Cells and Epithelial-Mesenchymal Transition-type Cells in Pancreatic Cancer. Front. Oncol. 2, 209. 10.3389/fonc.2012.00209 23316479 PMC3539658

[B51] Karamitopoulou E. Zlobec I. Born D. Kondi-Pafiti A. Lykoudis P. Mellou A. (2013). Tumour Budding Is a strong and Independent Prognostic Factor in Pancreatic Cancer. Eur. J. Cancer 49, 1032–1039. 10.1016/j.ejca.2012.10.022 23177090

[B52] Kimmel R. A. Meyer D. (2010). Molecular Regulation of Pancreas Development in Zebrafish. Methods Cel Biol 100, 261–280. 10.1016/B978-0-12-384892-5.00010-4 21111221

[B53] Kolodziejczyk A. A. Kim J. K. Svensson V. Marioni J. C. Teichmann S. A. (2015). The Technology and Biology of Single-Cell RNA Sequencing. Mol. Cel 58, 610–620. 10.1016/j.molcel.2015.04.005 26000846

[B54] Kulemann B. Pitman M. B. Liss A. S. Valsangkar N. Fernández-del Castillo C. Lillemoe K. D. (2015). Circulating Tumor Cells Found in Patients with Localized and Advanced Pancreatic Cancer. Pancreas 44, 547–550. 10.1097/MPA.0000000000000324 25822154

[B55] La Manno G. Soldatov R. Zeisel A. Braun E. Hochgerner H. Petukhov V. (2018). RNA Velocity of Single Cells. Nature 560, 494–498. 10.1038/s41586-018-0414-6 30089906 PMC6130801

[B56] Laklai H. Miroshnikova Y. A. Pickup M. W. Collisson E. A. Kim G. E. Barrett A. S. (2016). Genotype Tunes Pancreatic Ductal Adenocarcinoma Tissue Tension to Induce Matricellular Fibrosis and Tumor Progression. Nat. Med. 22, 497–505. 10.1038/nm.4082 27089513 PMC4860133

[B57] Lam C. J. Cox A. R. Jacobson D. R. Rankin M. M. Kushner J. A. (2018). Highly Proliferative α-Cell-Related Islet Endocrine Cells in Human Pancreata. Diabetes 67, 674–686. 10.2337/db17-1114 29326366 PMC5860854

[B58] Lapin M. Tjensvoll K. Oltedal S. Javle M. Smaaland R. Gilje B. (2017). Single-cell mRNA Profiling Reveals Transcriptional Heterogeneity Among Pancreatic Circulating Tumour Cells. BMC Cancer 17, 390. 10.1186/s12885-017-3385-3 28569190 PMC5452374

[B59] Lawlor N. George J. Bolisetty M. Kursawe R. Sun L. Sivakamasundari V. (2017a). Single-cell Transcriptomes Identify Human Islet Cell Signatures and Reveal Cell-type-specific Expression Changes in Type 2 Diabetes. Genome Res. 27, 208–222. 10.1101/gr.212720.116 27864352 PMC5287227

[B60] Lawlor N. Khetan S. Ucar D. Stitzel M. L. (2017b). Genomics of Islet (Dys)function and Type 2 Diabetes. Trends Genet. 33, 244–255. 10.1016/j.tig.2017.01.010 28245910 PMC5458785

[B61] Lazova R. Laberge G. S. Duvall E. Spoelstra N. Klump V. Sznol M. (2013). A Melanoma Brain Metastasis with a Donor-Patient Hybrid Genome Following Bone Marrow Transplantation: First Evidence for Fusion in Human Cancer. PLoS One 8, e66731. 10.1371/journal.pone.0066731 23840523 PMC3694119

[B62] Le D. T. Uram J. N. Wang H. Bartlett B. R. Kemberling H. Eyring A. D. (2015). PD-1 Blockade in Tumors with Mismatch-Repair Deficiency. N. Engl. J. Med. 372, 2509–2520. 10.1056/NEJMoa1500596 26028255 PMC4481136

[B63] Li J. Klughammer J. Farlik M. Penz T. Spittler A. Barbieux C. (2016a). Single‐cell Transcriptomes Reveal Characteristic Features of Human Pancreatic Islet Cell Types. EMBO Rep. 17, 178–187. 10.15252/embr.201540946 26691212 PMC4784001

[B64] Li W. Cerise J. E. Yang Y. Han H. (2017). Application of T-SNE to Human Genetic Data. J. Bioinform. Comput. Biol. 15, 1750017. 10.1142/S0219720017500172 28718343

[B65] Li Y. Zhang Y. Yao Z. Li S. Yin Z. Xu M. (2016b). Forkhead Box Q1: A Key Player in the Pathogenesis of Tumors (Review). Int. J. Oncol. 49, 51–58. 10.3892/ijo.2016.3517 27176124

[B66] Ligorio M. Sil S. Malagon-Lopez J. Nieman L. T. Misale S. Di Pilato M. (2019). Stromal Microenvironment Shapes the Intratumoral Architecture of Pancreatic Cancer. Cell 178, 160–175 e127. 10.1016/j.cell.2019.05.012 31155233 PMC6697165

[B67] Liu M. Haataja L. Wright J. Wickramasinghe N. P. Hua Q.-X. Phillips N. F. (2010). Mutant INS-Gene Induced Diabetes of Youth: Proinsulin Cysteine Residues Impose Dominant-Negative Inhibition on Wild-type Proinsulin Transport. PLoS One 5, e13333. 10.1371/journal.pone.0013333 20948967 PMC2952628

[B68] Liu R.-Y. Zeng Y. Lei Z. Wang L. Yang H. Liu Z. (2014). JAK/STAT3 Signaling Is Required for TGF-β-Induced Epithelial-Mesenchymal Transition in Lung Cancer Cells. Int. J. Oncol. 44, 1643–1651. 10.3892/ijo.2014.2310 24573038

[B69] Luo Q. Fu Q. Zhang X. Zhang H. Qin T. (2020). Application of Single-Cell RNA Sequencing in Pancreatic Cancer and the Endocrine Pancreas. Adv. Exp. Med. Biol. 1255, 143–152. 10.1007/978-981-15-4494-1_12 32949397

[B70] Lytle N. K. Ferguson L. P. Rajbhandari N. Gilroy K. Fox R. G. Deshpande A. (2019). A Multiscale Map of the Stem Cell State in Pancreatic Adenocarcinoma. Cell 177, 572–586 e522. 10.1016/j.cell.2019.03.010 30955884 PMC6711371

[B71] Ma L. Zheng J. (2018). Single-cell Gene Expression Analysis Reveals β-cell Dysfunction and Deficit Mechanisms in Type 2 Diabetes. BMC Bioinformatics 19, 515. 10.1186/s12859-018-2519-1 30598071 PMC6311914

[B72] Meier J. J. Bonadonna R. C. (2013). Role of Reduced -Cell Mass versus Impaired -Cell Function in the Pathogenesis of Type 2 Diabetes. Diabetes Care 36 (Suppl. 2), S113–S119. 10.2337/dcS13-2008 23882035 PMC3920783

[B73] Menheniott T. R. O’Connor L. Chionh Y. T. Däbritz J. Scurr M. Rollo B. N. (2016). Loss of Gastrokine-2 Drives Premalignant Gastric Inflammation and Tumor Progression. J. Clin. Invest. 126, 1383–1400. 10.1172/JCI82655 26974160 PMC4811116

[B74] Menke A. Casagrande S. Geiss L. Cowie C. C. (2015). Prevalence of and Trends in Diabetes Among Adults in the United States, 1988-2012. JAMA 314, 1021–1029. 10.1001/jama.2015.10029 26348752

[B75] Migliorini A. Bader E. Lickert H. (2014). Islet Cell Plasticity and Regeneration. Mol. Metab. 3, 268–274. 10.1016/j.molmet.2014.01.010 24749056 PMC3986629

[B76] Millman J. R. Xie C. Van Dervort A. Gürtler M. Pagliuca F. W. Melton D. A. (2016). Generation of Stem Cell-Derived β-cells from Patients with Type 1 Diabetes. Nat. Commun. 7, 11463. 10.1038/ncomms11463 27163171 PMC4866045

[B77] Moffitt R. A. Marayati R. Flate E. L. Volmar K. E. Loeza S. G. H. Hoadley K. A. (2015). Virtual Microdissection Identifies Distinct Tumor- and Stroma-specific Subtypes of Pancreatic Ductal Adenocarcinoma. Nat. Genet. 47, 1168–1178. 10.1038/ng.3398 26343385 PMC4912058

[B78] Muraro M. J. Dharmadhikari G. Grün D. Groen N. Dielen T. Jansen E. (2016). A Single-Cell Transcriptome Atlas of the Human Pancreas. Cel Syst. 3, 385–394 e383. 10.1016/j.cels.2016.09.002 PMC5092539 27693023

[B79] Nomiyama T. Perez-Tilve D. Ogawa D. Gizard F. Zhao Y. Heywood E. B. (2007). Osteopontin Mediates Obesity-Induced Adipose Tissue Macrophage Infiltration and Insulin Resistance in Mice. J. Clin. Invest. 117, 2877–2888. 10.1172/JCI31986 17823662 PMC1964510

[B80] Öhlund D. Handly-Santana A. Biffi G. Elyada E. Almeida A. S. Ponz-Sarvise M. (2017). Distinct Populations of Inflammatory Fibroblasts and Myofibroblasts in Pancreatic Cancer. J. Exp. Med. 214, 579–596. 10.1084/jem.20162024 28232471 PMC5339682

[B81] Olsson A. Venkatasubramanian M. Chaudhri V. K. Aronow B. J. Salomonis N. Singh H. (2016). Single-cell Analysis of Mixed-Lineage States Leading to a Binary Cell Fate Choice. Nature 537, 698–702. 10.1038/nature19348 27580035 PMC5161694

[B82] Özdemir B. C. Pentcheva-Hoang T. Carstens J. L. Zheng X. Wu C.-C. Simpson T. R. (2014). Depletion of Carcinoma-Associated Fibroblasts and Fibrosis Induces Immunosuppression and Accelerates Pancreas Cancer with Reduced Survival. Cancer Cell 25, 719–734. 10.1016/j.ccr.2014.04.005 24856586 PMC4180632

[B83] Pagliuca F. W. Millman J. R. Gürtler M. Segel M. Van Dervort A. Ryu J. H. (2014). Generation of Functional Human Pancreatic β Cells *In Vitro* . Cell 159, 428–439. 10.1016/j.cell.2014.09.040 25303535 PMC4617632

[B84] Patel M. B. Pothula S. P. Xu Z. Lee A. K. Goldstein D. Pirola R. C. (2014). The Role of the Hepatocyte Growth Factor/c-MET Pathway in Pancreatic Stellate Cell-Endothelial Cell Interactions: Antiangiogenic Implications in Pancreatic Cancer. Carcinogenesis 35, 1891–1900. 10.1093/carcin/bgu122 24876152

[B85] Poudel A. Savari O. Striegel D. A. Periwal V. Taxy J. Millis J. M. (2015). Beta-cell Destruction and Preservation in Childhood and Adult Onset Type 1 Diabetes. Endocrine 49, 693–702. 10.1007/s12020-015-0534-9 25605478 PMC4511725

[B86] Powell A. E. Anderson E. C. Davies P. S. Silk A. D. Pelz C. Impey S. (2011). Fusion between Intestinal Epithelial Cells and Macrophages in a Cancer Context Results in Nuclear Reprogramming. Cancer Res. 71, 1497–1505. 10.1158/0008-5472.CAN-10-3223 21303980 PMC3079548

[B87] Principe D. R. Diaz A. M. Torres C. Mangan R. J. Decant B. Mckinney R. (2017). TGFβ Engages MEK/ERK to Differentially Regulate Benign and Malignant Pancreas Cell Function. Oncogene 36, 4336–4348. 10.1038/onc.2016.500 28368414 PMC5537609

[B88] Qiu W.-L. Zhang Y.-W. Feng Y. Li L.-C. Yang L. Xu C.-R. (2017). Deciphering Pancreatic Islet β Cell and α Cell Maturation Pathways and Characteristic Features at the Single-Cell Level. Cel Metab. 25, 1194–1205 e1194. 10.1016/j.cmet.2017.04.003 28467935

[B89] Rahier J. Guiot Y. Goebbels R. M. Sempoux C. Henquin J. C. (2008). Pancreatic β-cell Mass in European Subjects with Type 2 Diabetes. Diabetes Obes. Metab. 10 (Suppl. 4), 32–42. 10.1111/j.1463-1326.2008.00969.x 18834431

[B90] Ranjan A. K. Joglekar M. V. Hardikar A. (2009). Endothelial Cells in Pancreatic Islet Development and Function. Islets 1, 2–9. 10.4161/isl.1.1.9054 21084843

[B91] Rhim A. D. Oberstein P. E. Thomas D. H. Mirek E. T. Palermo C. F. Sastra S. A. (2014). Stromal Elements Act to Restrain, rather Than Support, Pancreatic Ductal Adenocarcinoma. Cancer Cell 25, 735–747. 10.1016/j.ccr.2014.04.021 24856585 PMC4096698

[B92] Roscioni S. S. Migliorini A. Gegg M. Lickert H. (2016). Impact of Islet Architecture on β-cell Heterogeneity, Plasticity and Function. Nat. Rev. Endocrinol. 12, 695–709. 10.1038/nrendo.2016.147 27585958

[B93] Rotinen M. You S. Yang J. Coetzee S. G. Reis-Sobreiro M. Huang W.-C. (2018). ONECUT2 Is a Targetable Master Regulator of Lethal Prostate Cancer that Suppresses the Androgen axis. Nat. Med. 24, 1887–1898. 10.1038/s41591-018-0241-1 30478421 PMC6614557

[B94] Russ H. A. Parent A. V. Ringler J. J. Hennings T. G. Nair G. G. Shveygert M. (2015). Controlled Induction of Human Pancreatic Progenitors Produces Functional Beta‐like Cells *In Vitro* . EMBO J. 34, 1759–1772. 10.15252/embj.201591058 25908839 PMC4516429

[B95] Saadatpour A. Lai S. Guo G. Yuan G.-C. (2015). Single-Cell Analysis in Cancer Genomics. Trends Genet. 31, 576–586. 10.1016/j.tig.2015.07.003 26450340 PMC5282606

[B96] Sandberg R. (2014). Entering the Era of Single-Cell Transcriptomics in Biology and Medicine. Nat. Methods 11, 22–24. 10.1038/nmeth.2764 24524133

[B97] Scheuner D. Kaufman R. J. (2008). The Unfolded Protein Response: A Pathway that Links Insulin Demand with β-Cell Failure and Diabetes. Endocr. Rev. 29, 317–333. 10.1210/er.2007-0039 18436705 PMC2528859

[B98] Schlesinger Y. Yosefov-Levi O. Kolodkin-Gal D. Granit R. Z. Peters L. Kalifa R. (2020). Single-cell Transcriptomes of Pancreatic Preinvasive Lesions and Cancer Reveal Acinar Metaplastic Cells' Heterogeneity. Nat. Commun. 11, 4516. 10.1038/s41467-020-18207-z 32908137 PMC7481797

[B99] Segerstolpe Å. Palasantza A. Eliasson P. Andersson E.-M. Andréasson A.-C. Sun X. (2016). Single-Cell Transcriptome Profiling of Human Pancreatic Islets in Health and Type 2 Diabetes. Cel Metab. 24, 593–607. 10.1016/j.cmet.2016.08.020 PMC5069352 27667667

[B100] Shapiro E. Biezuner T. Linnarsson S. (2013). Single-cell Sequencing-Based Technologies Will Revolutionize Whole-Organism Science. Nat. Rev. Genet. 14, 618–630. 10.1038/nrg3542 23897237

[B101] Shapiro J. Bruni A. Pepper A. R. Gala-Lopez B. Abualhassan N. S. (2014). Islet Cell Transplantation for the Treatment of Type 1 Diabetes: Recent Advances and Future Challenges. Dmso 7, 211–223. 10.2147/DMSO.S50789 PMC4075233 25018643

[B102] Sharon N. Chawla R. Mueller J. Vanderhooft J. Whitehorn L. J. Rosenthal B. (2019). A Peninsular Structure Coordinates Asynchronous Differentiation with Morphogenesis to Generate Pancreatic Islets. Cell 176, 790–804 e713. 10.1016/j.cell.2018.12.003 30661759 PMC6705176

[B103] Shekhar K. Lapan S. W. Whitney I. E. Tran N. M. Macosko E. Z. Kowalczyk M. (2016). Comprehensive Classification of Retinal Bipolar Neurons by Single-Cell Transcriptomics. Cell 166, 1308–1323 e1330. 10.1016/j.cell.2016.07.054 27565351 PMC5003425

[B104] Simpson L. A. Miller W. C. Eng J. J. (2011). Effect of Stroke on Fall Rate, Location and Predictors: a Prospective Comparison of Older Adults with and without Stroke. PLoS One 6, e19431. 10.1371/journal.pone.0019431 21559367 PMC3084849

[B105] Stegle O. Teichmann S. A. Marioni J. C. (2015). Computational and Analytical Challenges in Single-Cell Transcriptomics. Nat. Rev. Genet. 16, 133–145. 10.1038/nrg3833 25628217

[B106] Steiner D. J. Kim A. Miller K. Hara M. (2010). Pancreatic Islet Plasticity: Interspecies Comparison of Islet Architecture and Composition. Islets 2, 135–145. 10.4161/isl.2.3.11815 20657742 PMC2908252

[B107] Steward M. C. Ishiguro H. Case R. M. (2005). Mechanisms of Bicarbonate Secretion in the Pancreatic Duct. Annu. Rev. Physiol. 67, 377–409. 10.1146/annurev.physiol.67.031103.153247 15709963

[B108] Surguchov A. (2020). Caveolin: A New Link between Diabetes and AD. Cell Mol Neurobiol 40, 1059–1066. 10.1007/s10571-020-00796-4 31974905 PMC11448860

[B109] Svensson V. Natarajan K. N. Ly L.-H. Miragaia R. J. Labalette C. Macaulay I. C. (2017). Power Analysis of Single-Cell RNA-Sequencing Experiments. Nat. Methods 14, 381–387. 10.1038/nmeth.4220 28263961 PMC5376499

[B110] Tang F. Barbacioru C. Wang Y. Nordman E. Lee C. Xu N. (2009). mRNA-Seq Whole-Transcriptome Analysis of a Single Cell. Nat. Methods 6, 377–382. 10.1038/nmeth.1315 19349980

[B111] Tang F. Lao K. Surani M. A. (2011). Development and Applications of Single-Cell Transcriptome Analysis. Nat. Methods 8, S6–S11. 10.1038/nmeth.1557 21451510 PMC3408593

[B112] Tang L.-Y. Heller M. Meng Z. Yu L.-R. Tang Y. Zhou M. (2017). Transforming Growth Factor-β (TGF-β) Directly Activates the JAK1-STAT3 Axis to Induce Hepatic Fibrosis in Coordination with the SMAD Pathway. J. Biol. Chem. 292, 4302–4312. 10.1074/jbc.M116.773085 28154170 PMC5354477

[B113] Thorel F. Népote V. Avril I. Kohno K. Desgraz R. Chera S. (2010). Conversion of Adult Pancreatic α-cells to β-cells after Extreme β-cell Loss. Nature 464, 1149–1154. 10.1038/nature08894 20364121 PMC2877635

[B114] Ting D. T. Wittner B. S. Ligorio M. Vincent Jordan N. Shah A. M. Miyamoto D. T. (2014). Single-cell RNA Sequencing Identifies Extracellular Matrix Gene Expression by Pancreatic Circulating Tumor Cells. Cel Rep. 8, 1905–1918. 10.1016/j.celrep.2014.08.029 PMC4230325 25242334

[B115] Unanue E. R. (2016). Macrophages in Endocrine Glands, with Emphasis on Pancreatic Islets. Microbiol. Spectr. 4. 10.1128/microbiolspec.MCHD-0048-2016 PMC5240809 28084197

[B116] Van Der Meulen T. Mawla A. M. Digruccio M. R. Adams M. W. Nies V. Dólleman S. (2017). Virgin Beta Cells Persist throughout Life at a Neogenic Niche within Pancreatic Islets. Cel Metab. 25, 911–926 e916. 10.1016/j.cmet.2017.03.017 PMC8586897 28380380

[B117] Villani V. Thornton M. E. Zook H. N. Crook C. J. Grubbs B. H. Orlando G. (2019). SOX9+/PTF1A+ Cells Define the Tip Progenitor Cells of the Human Fetal Pancreas of the Second Trimester. STEM CELLS Translational Med. 8, 1249–1264. 10.1002/sctm.19-0231 PMC6877773 31631582

[B118] Waddell N. Pajic M. Pajic M. Patch A.-M. Chang D. K. Kassahn K. S. (2015). Whole Genomes Redefine the Mutational Landscape of Pancreatic Cancer. Nature 518, 495–501. 10.1038/nature14169 25719666 PMC4523082

[B119] Wang J. Osei K. (2011). Proinsulin Maturation Disorder Is a Contributor to the Defect of Subsequent Conversion to Insulin in β-cells. Biochem. Biophysical Res. Commun. 411, 150–155. 10.1016/j.bbrc.2011.06.119 21723250

[B120] Wang X. Misawa R. Zielinski M. C. Cowen P. Jo J. Periwal V. (2013). Regional Differences in Islet Distribution in the Human Pancreas - Preferential Beta-Cell Loss in the Head Region in Patients with Type 2 Diabetes. PLoS One 8, e67454. 10.1371/journal.pone.0067454 23826303 PMC3691162

[B121] Wang Y. J. Golson M. L. Schug J. Traum D. Liu C. Vivek K. (2016a). Single-Cell Mass Cytometry Analysis of the Human Endocrine Pancreas. Cel Metab. 24, 616–626. 10.1016/j.cmet.2016.09.007 PMC5123805 27732837

[B122] Wang Y. J. Kaestner K. H. (2019). Single-Cell RNA-Seq of the Pancreatic Islets-Aa Promise Not yet Fulfilled? Cel. Metab. 29, 539–544. 10.1016/j.cmet.2018.11.016 PMC6402960 30581120

[B123] Wang Y. J. Schug J. Won K.-J. Liu C. Naji A. Avrahami D. (2016b). Single-Cell Transcriptomics of the Human Endocrine Pancreas. Diabetes 65, 3028–3038. 10.2337/db16-0405 27364731 PMC5033269

[B124] Wang Y. Wu C. Zhang C. Li Z. Zhu T. Chen J. (2018). TGF-β-induced STAT3 Overexpression Promotes Human Head and Neck Squamous Cell Carcinoma Invasion and Metastasis through malat1/miR-30a Interactions. Cancer Lett. 436, 52–62. 10.1016/j.canlet.2018.08.009 30118844

[B125] Whitcomb D. C. Lowe M. E. (2007). Human Pancreatic Digestive Enzymes. Dig. Dis. Sci. 52, 1–17. 10.1007/s10620-006-9589-z 17205399

[B126] Witkiewicz A. K. Mcmillan E. A. Balaji U. Baek G. Lin W.-C. Mansour J. (2015). Whole-exome Sequencing of Pancreatic Cancer Defines Genetic Diversity and Therapeutic Targets. Nat. Commun. 6, 6744. 10.1038/ncomms7744 25855536 PMC4403382

[B127] Wollny D. Zhao S. Everlien I. Lun X. Brunken J. Brüne D. (2016). Single-Cell Analysis Uncovers Clonal Acinar Cell Heterogeneity in the Adult Pancreas. Dev. Cel 39, 289–301. 10.1016/j.devcel.2016.10.002 27923766

[B128] Xin Y. Dominguez Gutierrez G. Okamoto H. Kim J. Lee A.-H. Adler C. (2018). Pseudotime Ordering of Single Human β-Cells Reveals States of Insulin Production and Unfolded Protein Response. Diabetes 67, 1783–1794. 10.2337/db18-0365 29950394

[B129] Xin Y. Kim J. Okamoto H. Ni M. Wei Y. Adler C. (2016). RNA Sequencing of Single Human Islet Cells Reveals Type 2 Diabetes Genes. Cel Metab. 24, 608–615. 10.1016/j.cmet.2016.08.018 27667665

[B130] Xue Z. Huang K. Cai C. Cai L. Jiang C.-y. Feng Y. (2013). Genetic Programs in Human and Mouse Early Embryos Revealed by Single-Cell RNA Sequencing. Nature 500, 593–597. 10.1038/nature12364 23892778 PMC4950944

[B131] Yan L. Yang M. Guo H. Yang L. Wu J. Li R. (2013). Single-cell RNA-Seq Profiling of Human Preimplantation Embryos and Embryonic Stem Cells. Nat. Struct. Mol. Biol. 20, 1131–1139. 10.1038/nsmb.2660 23934149

[B132] Yoon K. H. Ko S. H. Cho J. H. Lee J. M. Ahn Y. B. Song K. H. (2003). Selective β-Cell Loss and α-Cell Expansion in Patients with Type 2 Diabetes Mellitus in Korea. J. Clin. Endocrinol. Metab. 88, 2300–2308. 10.1210/jc.2002-020735 12727989

[B133] Yu X.-X. Xu C.-R. (2020). Understanding Generation and Regeneration of Pancreatic β Cells from a Single-Cell Perspective. Development 147. 10.1242/dev.179051 32280064

[B134] Yu X. X. Qiu W. L. Yang L. Zhang Y. He M. Y. Li L. C. (2019). Defining Multistep Cell Fate Decision Pathways during Pancreatic Development at Single‐cell Resolution. EMBO J. 38. 10.15252/embj.2018100164 PMC6463266 30737258

[B135] Zeisel A. Muñoz-Manchado A. B. Codeluppi S. Lönnerberg P. La Manno G. Juréus A. (2015). Cell Types in the Mouse Cortex and hippocampus Revealed by Single-Cell RNA-Seq. Science 347, 1138–1142. 10.1126/science.aaa1934 25700174

[B136] Zeng C. Mulas F. Sui Y. Guan T. Miller N. Tan Y. (2017). Pseudotemporal Ordering of Single Cells Reveals Metabolic Control of Postnatal β Cell Proliferation. Cel Metab. 25, 1160–1175 e1111. 10.1016/j.cmet.2017.04.014 PMC5501713 28467932

[B137] Zhao C. Xiao H. Wu X. Li C. Liang G. Yang S. (2015). Rational Combination of MEK Inhibitor and the STAT3 Pathway Modulator for the Therapy in K-Ras Mutated Pancreatic and colon Cancer Cells. Oncotarget 6, 14472–14487. 10.18632/oncotarget.3991 25961376 PMC4546480

